# The ZEB2‐dependent EMT transcriptional programme drives therapy resistance by activating nucleotide excision repair genes *ERCC1* and *ERCC4* in colorectal cancer

**DOI:** 10.1002/1878-0261.12965

**Published:** 2021-05-01

**Authors:** Rahul Sreekumar, Hajir Al‐Saihati, Muhammad Emaduddin, Karwan Moutasim, Massimiliano Mellone, Ashish Patel, Seval Kilic, Metin Cetin, Sule Erdemir, Marta Salgado Navio, Maria Antonette Lopez, Nathan Curtis, Tamer Yagci, John N. Primrose, Brendan D. Price, Geert Berx, Gareth J. Thomas, Eugene Tulchinsky, Alex Mirnezami, A. Emre Sayan

**Affiliations:** ^1^ Cancer Sciences Division University of Southampton UK; ^2^ College of Applied Medical Sciences University of Hafr Al‐Batin Saudi Arabia; ^3^ Department of Molecular Biology and Genetics Gebze Technical University Turkey; ^4^ Department of Surgery Southampton University Hospital NHS Trust UK; ^5^ Department of Radiation Oncology Dana‐Farber Cancer Institute Harvard Medical School Boston MA USA; ^6^ Molecular Cellular Oncology Lab Department for Biomedical Molecular Biology Ghent University Belgium; ^7^ Cancer Research Institute Ghent (CRIG) Belgium; ^8^ Department of Biomedical Sciences Nazarbayev University School of Medicine Astana Kazakhstan

**Keywords:** DNA repair, EMT, ERCC1, oxaliplatin, ZEB2

## Abstract

Resistance to adjuvant chemotherapy is a major clinical problem in the treatment of colorectal cancer (CRC). The aim of this study was to elucidate the role of an epithelial to mesenchymal transition (EMT)‐inducing protein, ZEB2, in chemoresistance of CRC, and to uncover the underlying mechanism. We performed IHC for ZEB2 and association analyses with clinical outcomes on primary CRC and matched CRC liver metastases in compliance with observational biomarker study guidelines. ZEB2 expression in primary tumours was an independent prognostic marker of reduced overall survival and disease‐free survival in patients who received adjuvant FOLFOX chemotherapy. ZEB2 expression was retained in 96% of liver metastases. The ZEB2‐dependent EMT transcriptional programme activated nucleotide excision repair (NER) pathway largely via upregulation of the *ERCC1* gene and other components in NER pathway, leading to enhanced viability of CRC cells upon oxaliplatin treatment. ERCC1‐overexpressing CRC cells did not respond to oxaliplatin *in vivo*, as assessed using a murine orthotopic model in a randomised and blinded preclinical study. Our findings show that ZEB2 is a biomarker of tumour response to chemotherapy and risk of recurrence in CRC patients. We propose that the ZEB2‐ERCC1 axis is a key determinant of chemoresistance in CRC.

## Graphical abstract

Here, we show that the expression of ZEB2 marks reduced overall and disease‐free survival in primary and secondary colorectal cancers (CRCs). ZEB2 overexpression promoted chemoresistance to oxaliplatin *in vitro* and *in vivo* by transcriptionally activating nucleotide excision repair genes ERCC1 and ERCC4. Overall ZEB2 is a promising biomarker predicting both therapy response and metastatic ability of CRCs.

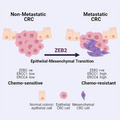

**Abbreviations**
5‐FU: 5‐Fluorouracil
CRC: colorectal cancer
DOX: doxycycline
DSB: double‐strand break
EMT: epithelial–mesenchymal transition
IHC: Immunohistochemistry
MET: mesenchymal–epithelial transition
NER: nucleotide excision repair

## 1 Introduction

Colorectal cancer (CRC) is the second most common cause of cancer‐associated mortality in Europe and a key public health issue [1]. Recurrence is the principle cause of mortality, observed in up to 30% at presentation and develop in 50% after curative surgery [2]. The majority of patients with recurrent disease are incurable and experience a median survival of < 3 years [3]. Surgical resection combined with DNA damaging agent, such as 5‐fluorouracil (5‐FU), irinotecan and oxaliplatin, based chemotherapeutic strategies (FOLFOX or FOLFIRI), with or without addition of biological agents remains the standard of care in high‐risk patients. Majority of patients, however, fail to respond to treatment and can suffer side effects without therapeutic benefit [4]. Despite the drive towards personalised care, the only biomarker in standard clinical use is KRAS mutation status which predicts response to EGFR inhibitors such as cetuximab [5]. Nevertheless, this example provides proof of principle that a mechanistic understanding of CRC biology can be translated to improved patient outcomes and highlights the pressing requirement for the identification of new predictive biomarkers of therapy response.

Epithelial–mesenchymal transition (EMT) is a conserved epigenetic programme that generates mesenchymal cells from epithelial sheets [6]. EMT is induced by a variety of signalling pathways leading to repression of epithelial and activation of mesenchymal genes [7, 8, 9]. The cardinal features of EMT also include acquisition of stem cell properties, increased motility and apoptosis resistance [10, 11]. Transcription factors that belong to Twist, SNAI and ZEB families (EMT‐TFs) execute EMT in cancer and have attracted attention due to their significant association with metastatic capability and chemoresistance [12].

The association between EMT, poor oncological outcomes and treatment resistance has been highlighted in many solid tumours [11]. Earlier studies described a link between drug resistance and EMT by incubating epithelial carcinoma cells with DNA damaging agents for extended periods and reporting the mesenchymal morphology of the selected (chemoresistant) cells [13]. In line with these *in vitro* observations, molecular stratification of CRC patients revealed patients displaying EMT gene expression respond poorly to adjuvant chemotherapy, experience earlier recurrence and reduced survival [14]. Despite these compelling observations, the cellular mechanisms driving EMT‐induced chemoresistance are poorly understood. Increased drug efflux, improved DNA repair, rewiring of cellular signalling, attenuated DNA damage response and pro‐apoptotic signalling have been suggested as contributing factors [6, 11, 15, 16, 17].

The function of ZEB2 has been sparsely studied in CRC, especially in the context of chemotherapy response. Here, we report nuclear ZEB2 immuno‐expression as a marker of poor response to adjuvant FOLFOX chemotherapy. CRC cells expressing ZEB2 undergo EMT and became resistant to oxaliplatin and 5‐FU, compounds administered in the FOLFOX regime to treat CRC patients. Critical components of the nucleotide excision repair (NER) pathway, such as ERCC1 and ERCC4, are induced upon ZEB2 expression. High ERCC1 abundance in CRC cells enhanced kinetics of oxaliplatin‐induced DNA crosslink clearance, thus promoting enhanced DNA repair and resistance to apoptosis both *in vitro* and *in vivo*. Taken together, these findings clarify the mechanism of ZEB2‐induced chemoresistance and suggest nuclear ZEB2 may have clinical utility in predicting recurrence and response to oxaliplatin‐based chemotherapy regimes in CRC.

## 2 Materials and methods

### 2.1 Patient material and analysis of ZEB2 immuno‐expression

All patients were recruited as part of an ongoing prospective UK National Institute of Health Research Clinical Research Network study (UK‐CRNID6067; NCT03309722) investigating the molecular pathology of CRC and designed to identify novel biomarkers. Details from this ongoing study have been previously described [18]; further details and other results have been previously described [18, 19]. Study oversight activities and monitoring were performed at an independent clinical research organisation. All patients provided written informed consent, and the regional research ethics committee approved the study (10/H0504/32) in line with ethical standards set by Helsinki Declaration. Written consent (to present data in anonymised form as presented in this study) is obtained from all patients. Following recruitment and surgery, tissue samples were deposited in a UK Human Tissue Act approved tumour bank. Pathological verification of diagnosis and staging was in accordance with the Association of Coloproctology of Great Britain and Ireland guidelines. Information relating to patient demographics, preoperative risk, imaging, surgery, pathological features, post‐op management and oncological outcomes was prospectively collected. For the present study, only patients who completed a full course of chemotherapy without dose reductions were evaluated. Exclusion criteria included evidence of a hereditary tumour, presence of multiple tumours, and tumours with histologically identified extensive necrosis. Paraffin‐embedded tissue specimens were retrieved for all patients in the present study. All IHC was conducted at the histochemistry research unit at the University of Southampton using an automated immunostaining device (Autostainer XL; Leica, Milton Keynes, UK). Stained sections were assessed for the presence of nuclear ZEB2 expression in neoplastic and normal tissue. Two independent pathologists blinded to the clinical details scored the sections as ZEB2‐positive or ZEB2‐negative using previously established and validated scoring criteria [15, 20] and rereviewed if there was a disparity in scoring. The scoring criteria are briefly nuclear positive staining with at least 10% cells of a tumour. Detailed validation of ZEB2 antibody in different techniques including IHC could be seen in Ref. [3, 4, 6]. ZEB2 expression was correlated to oncological outcomes to evaluate its role as a prognostic or predictive biomarker. All results have been reported in line with reporting standards for biomarker development proposed by REMARK guidelines. A REMARK biomarker standards profile for the present study is provided in Table S3.

### 2.2 Cell culture and transfections

DLD1, CT26, SW480, SW620, Colo205, Caco2, HT29, Lovo and RKO were purchased from ATCC, subjected to annual STR analysis and routinely screened for mycoplasma contamination (MycoAlert; Lonza, Slough, UK). Cells were propagated in Dulbecco's Modified Eagle's medium supplemented with 10% FCS, penicillin/streptomycin (50 U·mL^−1^) and 2 mm l‐glutamine in a humidified CO_2_ (5%) incubator. DLD1‐ZEB2 and A431‐ZEB2 cells have been previously described [21]. ZEB2 expression was induced by culturing DLD1‐ZEB2 cells in the presence of doxycycline (DOX) (2 μg·mL^−1^; Sigma, Gillingham, UK) for 3 days. All cell lines used, apart from CT26 (mouse primary CRC), were from patients with primary CRC. Transfections were performed using Lipofectamine 3000 reagent (Invitrogen, Oxford, UK). Where necessary, cells were treated with oxaliplatin (Hospira, Maidenhead, UK), 5‐FU (Sigma) and doxorubicin (Sigma). Small hairpin RNA constructs (control and two validated targeting human and mouse ERCC1 (cat no: SI02663430) and ERCC4 (cat no: SI03095883)) were purchased from Qiagen (Manchester, UK). ZEB2 siRNA targeting both human and mouse transcript was used as described before [22].

### 2.3 Generation of stable clones

DLD1 cells were transfected with ERCC1‐mCherry or control (mCherry) constructs kindly provided by A. Sancar (University of North Carolina, USA). ERCC1‐mCherry and Control‐mCherry constructs were transfected into DLD1 cells. Cells were cultured in the presence of neomycin (400 μm), harvested and seeded in a 96‐well plate at the confluency of 0.8 cells per well. Wells with single cells were identified, propagated and ERCC1 expression validated by western blotting and/or mCherry fluorescence.

### 2.4 Assessment of apoptosis, drug uptake and viability

To observe effects of various drugs on cell viability, a microscopy‐based system [23] or crystal violet assay was used. Briefly, 10 000 cells were seeded in 48‐well plates and incubated with drugs (six concentrations in quadruplicate for *IC*50 assay). Eight hours after drug treatment, wells were washed and fresh media was added. After 48–96 h, cells were washed with PBS, fixed with ice‐cold acetone/methanol (50/50) and stained with DAPI (Molecular Probes, Oxford, UK). A semi‐automated system was used to take pictures from the centre of each well (UV channel, 10× magnification, Olympus‐CKX41), and the cell number was determined using ImageJ program as described before [23]. Intact nuclei are counted as ‘live cells’. For crystal violet assay, cell seeding and treatment were done as mentioned above. After that, cells were fixed with 4% formaldehyde and stained with 1% crystal violet solution (in 20% methanol) for 10 min. After three washes with PBS and a final wash with water, the plates were dried overnight at RT. Following day, crystal violet is solubilised with MA solution (10% methanol, 10% acetic acid) and OD was measured at 580 nm. The results were analysed using graphpad prism (v7, San Diego, CA, USA) to identify IC50 values.

For drug efflux assay, DLD‐ZEB2 (un‐induced and induced) cells in culture were treated with doxorubicin (0.5 μg·mL^−1^), for 1 h, washed with PBS and allowed to recover in fresh media for further 4 h. Cells were then trypsinised, collected and analysed using flow cytometry (FACScalibur; BD, Oxford, UK). Florescence emission (mean fluorescence intensity) was used to quantify drug uptake.

Apoptosis was quantitated by identification of phosphatidylserine externalisation and propidium iodide (PI) uptake. Briefly, cells were trypsinised and suspended in 500 μL of binding buffer (HEPES buffer: 10 mm HEPES (pH 7.4), 150 mm NaCl, 5 mm KCl, 5 mm MgCl_2_ and 1.8 mm CaCl) along with FITC conjugated Annexin V. Samples were incubated in the dark on ice for 30 min. Hundred microliter of 50 μg·mL^−1^ of PI was added and incubated for a further 10 min. All samples were analysed by flow cytometry and Annexin V‐high cells were considered apoptotic. Additionally, PARP cleavage was always assessed to validate the biochemical hallmarks of apoptosis.

### 2.5 Western blotting, immunofluorescence and expression analysis

Western blotting was performed as described previously [22]. Membranes were incubated with HRP‐conjugated secondary antibodies (Sigma), and the signal was detected using West Dura substrate (Pierce, Oxford, UK) and autoradiography films or imaging system (Bio‐Rad, Watford, UK). The primary antibodies used are as follows ZEB2 (in house, described in [15, 22]), vimentin (1/1000; Cell Signalling, London, UK), ERCC1 (1/1000; Cell Signalling), ERCC4 (1/1000; Cell Signalling), ERCC2 (XPD, 1/500; RnD systems), XPA (1/500; RnD systems, Oxford, UK), (E‐cadherin (1/1000; BD Transduction laboratories), PARP (1/1000; Cell signalling), total‐H2aX (1/1000; RnD systems) and beta‐actin (1/2000; Sigma). For immunofluorescence, cells were cultured overnight on glass coverslips and fixed in 3.7% formaldehyde in PBS for 30 min at 4 °C. Cells were then permeabilised with 0.5% Triton X‐100 in PBS for 5 min. Blocking was achieved using 2.5% BSA in PBS and 0.1% Tween 20. Cells were incubated with primary antibodies (E‐cadherin, (BD) and ZEB2 [15, 22]) for 2 h, washed and further incubated with Alexafluor‐488‐ or Alexafluor‐594‐conjugated secondary antibodies (Thermo Fisher, Oxford, UK). Images were captured using a Leica confocal microscope.

RNA extraction and cDNA synthesis have been performed as described before [15]. PCR was performed using primers designed using primer blast and purchased from Sigma. Primer sequences are provided in Table S7. GAPDH was used as an internal control. Syber green was used for qPCRs and qPCR array (DNA damage/repair, PAHS‐029ZA‐2; Qiagen). Lists of primers are presented in Supporting information.

### 2.6 Statistics and *in silico* analysis

ibm‐spss statistic (v22, Portsmouth, UK) was used to analyse survival analysis and clinicopathological correlations. Investigators blinded to patient outcome performed biomarker‐related scoring. Primary study endpoints were defined as time from the date of primary resection to the date of death (OS) or recurrence (DFS). Univariate analysis using log‐rank test and multivariable analysis using Cox regression were used to investigate the prognostic or predictive value of ZEB2. Kaplan–Meier survival estimation method, log‐rank test and hazard ratio tables were used to compare differences. Clinicopathological correlation analysis was undertaken using a chi‐squared test. Power calculations were performed using nQuery statistical software. Raw‐read counts from the CCLE‐CRC or GTEx53 cohorts of ExpressionAtlas database were loaded into edgeR software and heat maps were created using *pheatmap* add‐on. A *P* value of <0.05 was considered statistically significant. Graphical abstract was created with BioRender.com.

### 2.7 Cloning of ERCC1 regulatory region, generation of reporter constructs, transient transfection and luciferase reporter assay

The regulatory region of the *ERCC1* gene (n.t. −1980 to +765) was analysed for E‐box elements (CANNTG). Primers with restriction enzyme sites (HindI‐III/Sac‐I) were designed (Table S7) and segments of the *ERCC1* promoter (A and B) PCR‐amplified. Individual promoter DNA fragments were inserted into the PGL3‐basic vector to generate PGL3‐luc‐A, and PGL3‐luc‐B constructs and sequence verified. The dual‐Luciferase® reporter assay system (Promega; catalogue no: E1910, Southampton, UK) was used for the promoter reporter assay. DLD1‐ZEB2 cells were co‐transfected using Lipofectamine 3000 reagent with promoter constructs and renilla luciferase expression vector (internal control) and cultured in 96‐well plates. Cells were lysed by the application of PLB buffer for 15 min at room temperature. After lysis, LSA and SGS buffers were applied in turn and bioluminescence measured using a luminometer (ThermoFisher). Firefly Luciferase signal was measured to quantify ZEB2 promoter activity and normalised with renilla luminescence to control for transfection efficiency. The results were analysed using graphpad prism (v7).

### 2.8 Chromatin immunoprecipitation (ChIP)

ChIP experiments were performed using ChIP‐IT high‐sensitivity kit (ActiveMotif‐Catalogue no: 53040) following manufacturer’s guidelines. Briefly, un‐induced and induced cells were cultured in 10‐cm plates and fixed using 2% formaldehyde. Chromatin sonication was optimised by visualisation of DNA fragmentation after agarose gel electrophoresis. Chromatin was incubated with control IgG, RNA pol II (ActiveMotif ChIP‐IT® Control kit, catalogue no: 53010) or monoclonal anti‐c‐Myc (Sigma‐Aldrich) antibodies. ChIP grade anti‐c‐Myc antibody was used to pull down myc‐tagged ZEB2. Immunoprecipitation was carried out using protein‐G agarose beads. After protein digestion using proteinase K, PCR was performed as described previously [15]. The E‐box elements in *CDH1* regulatory regions were amplified as follows. E‐Boxes at positions −859 (E‐box1), −575 (E‐box2) and −399 (E‐box3) were amplified using CDH1‐F E‐box 1‐3 and CDH1‐R E‐box 1‐3 primers. The 4th and 5th E‐boxes at positions +39 and +89 were amplified using CDH1‐F E‐box 4‐5, CDH1‐R E‐box 4‐5 primer pair. ERCC1 regulatory regions (E‐boxes 1‐4) were amplified using ERCC1‐F1 EBOX 1‐4 and ERCC1‐R1 EBOX 1‐4 primer pair using PCR and presented on agarose gel. The E‐Boxes on ERCC1 promoter (E‐boxes 5–7) were amplified using primer pair ERCC1‐F1 EBOX 5‐7 and ERCC1‐R1 EBOX 5‐7 and qPCR. Coordinates of the E‐boxes are provided according to the transcription start site of the respected gene. List of primers, amplicon sizes and amplification protocol are presented in Table S7.

### 2.9 Slot blot assay

DLD‐ZEB2, ERCC1‐expressing wt‐DLD1 (DLD‐E1 and DLD‐E2) or control (DLD‐C1 and DLD‐C2) cell lines were treated with varying concentrations of oxaliplatin (1–24 h). GeneElute mammalian DNA purification kit (Sigma‐Aldrich) was used to extract genomic DNA and quantified using Nanodrop (ThermoFisher). Appropriate volume of DNA was diluted in 6X standard saline citrate (3 m NaCl; 300 mm sodium citrate; pH: 7.0) to load 1 μg onto a nitrocellulose membrane using a slot blot apparatus (Bio‐DOT‐SF; Bio‐Rad). The nitrocellulose membrane was baked at 80 °C for 2 h to crosslink DNA to the membrane. Immunoblotting was performed using anticisplatin DNA adduct antibody (clone ICR4; Sigma‐Aldrich) and visualised using Supersignal West Dura chemiluminescense detection kit (ThermoFisher), X‐ray film and developer. Equal loading of DNA was validated by staining the nitrocellulose membrane with SyberGreen gold (Invitrogen) and transilluminator (3UVP™; ThermoFisher).

### 2.10 *In vivo* studies

All *in vivo* experiments were approved by the local ethical committee and part of a UK Government Home Office project licence. DLD‐E2 (mCherry‐ERCC1) and DLD‐C2 (mCherry‐control) cells were used for *in vivo* experiments. Six‐ to 8‐week‐old female CD1‐nude mice (Charles River, Bristol, UK) were anaesthetised by inhalational anaesthesia prior to midline laparotomy and exteriorisation of the caecum for tumour implantations. Orthotopic implantation was conducted under magnified vision into the submucosal layer of the bowel. One million cells, suspended in Matrigel (1 : 1) were implanted per animal. After surgery, animals were recovered in a heated chamber. In total, 20 animals underwent orthotopic implantation of tumours. Primary tumours grew in all animals. After 4 weeks of recovery, mice were randomly allocated to treatment (oxaliplatin) or control arms and evaluated by individuals blinded to treatment regime received. The treatment arm was given intraperitoneal injection of oxaliplatin 5 mg·kg^−1^·week^−1^, while control animals were administered PBS in the same schedule. Animals were treated once a week for a 5‐week period. At week 9, animals were humanely culled in a CO_2_ chamber and their organs (caecum, lungs, liver and spleen) retrieved and weighed. Organs were also imaged and analysed using an IVIS Lumina III imaging unit (Perkin Elmer, Beaconsfield, UK). The unit of signal is set to average radiant efficiency (fluorescence emission radiance per incident excitation power) as recommended by the manufacturer. After analysis, organs were paraffin‐fixed, cut into sections and stained with haematoxylin and eosin to confirm imaging findings. Representative sections of the whole organ and all imaging were carefully analysed for the presence of primary tumour and metastases by specialist clinical pathologists and members of the team blinded to the treatment regime received. All mice were housed in a specific pathogen‐free facility in the University of Southampton and given a commercial basal diet and water *ad libitum*.

## 3 Results

### 3.1 Nuclear ZEB2 expression predicts poor response to FOLFOX chemotherapy

**Fig. 1:**
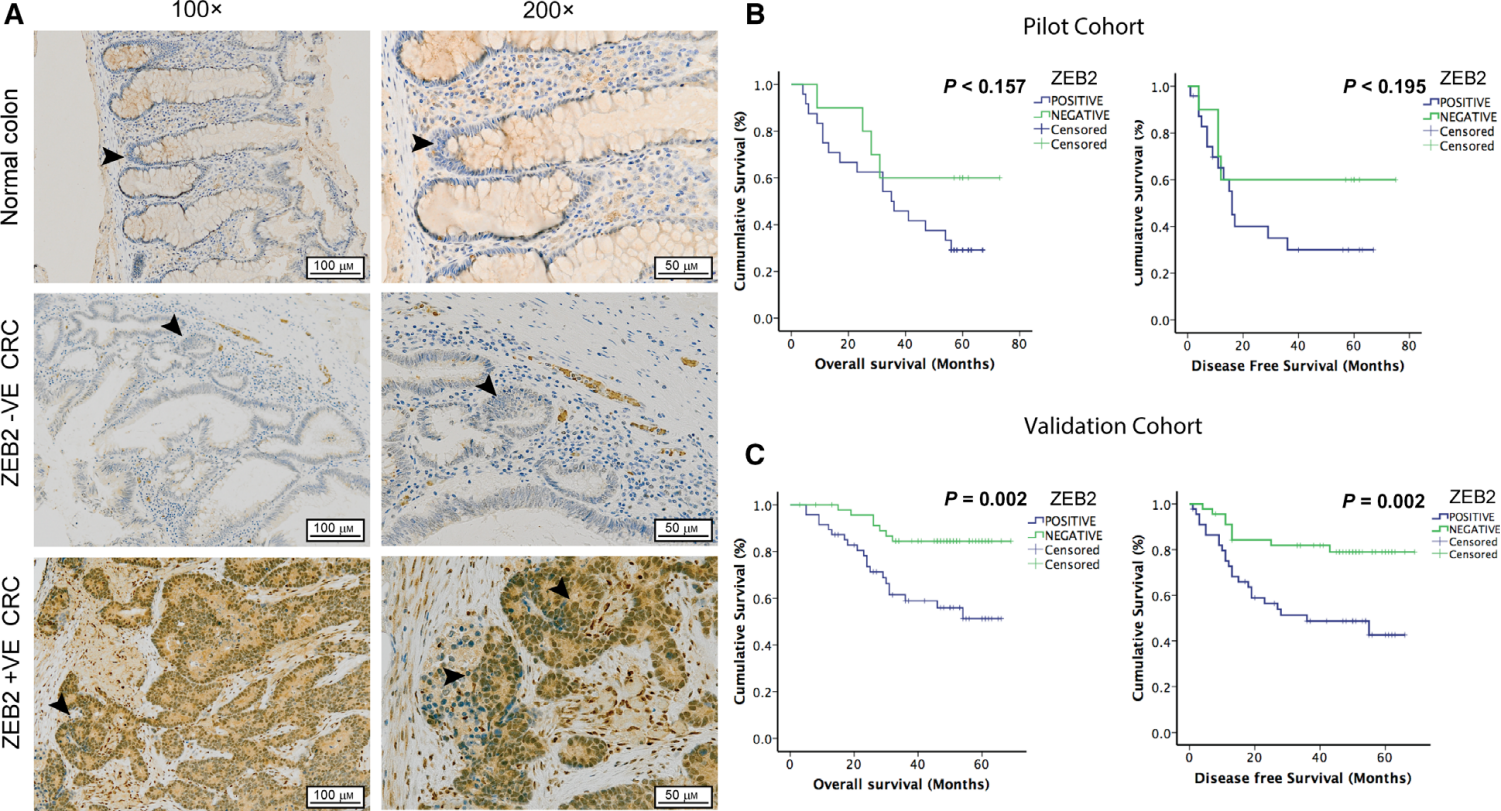
Nuclear ZEB2 predicts therapeutic efficacy of FOLFOX chemotherapy in patients with CRC. (A) Examples of representative IHC analyses of ZEB2 expression in normal colonic epithelium and primary CRC samples of patients who were administered adjuvant FOLFOX chemotherapy. The images exemplify ZEB2‐negative (−VE) and ZEB2‐positive (+VE) samples. Normal colonic cells and ZEB2 ‐VE tumours exhibit no positive staining other than in occasional stromal cells, whereas strong nuclear staining is evident in ZEB2 +VE tumours as marked by arrows. The scale bars are indicating 100 and 50 μm for 100× and 200× magnifications, respectively (B, C) Kaplan–Meier curves display OS and DFS for patients with ZEB2 –VE and ZEB2 +VE tumours in the pilot study (*n* = 34) (B) and validation cohort (*n* = 99) (C). Nuclear ZEB2 expression is associated with significantly reduced OS and DFS in the validation cohort (log‐rank, *P* = 0.002).

Previously, we published that ZEB2 induces resistance to DNA damage‐induced apoptosis in carcinoma cells [15]. We also reported that ZEB2 overexpression associates with early recurrence and reduced survival in CRC [24]. We aimed to validate these findings by assessing survival outcomes in CRC patients that received adjuvant chemotherapy. ZEB2 immunohistochemistry (IHC) and survival analysis were performed on a pilot cohort of 34 consecutive patients who completed FOLFOX regimen after surgical resection of primary CRC. ZEB2 scoring was performed using previously established criteria [20, 24]. ZEB2 was not detected in normal colonic epithelium whereas 71% (24/34) of the CRC specimens stained positive for nuclear ZEB2 (Fig. 1A). Survival analysis demonstrated a trend but nonsignificant reduction in mean overall survival (OS) of 15.6 months and disease‐free survival (DFS) of 19.5 months if ZEB2 was expressed (Fig 1B). Using this pilot data, a power calculation was undertaken to exclude the possibility of a type 2 error. We identified a minimum cohort size of 86 patients and 24 events as a requirement to achieve 80% power using a two‐sided test, at a significance of 5%, and assuming a hazard ratio of 3.0. Consequently, an independent validation cohort consisting of 99 further consecutive patients matching the previous inclusion/exclusion criteria were identified and analysed. A 15.9‐month reduction in mean OS (log‐rank, *P* < 0.002) and 19.5‐month reduction in mean DFS (log‐rank, *P* < 0.002) were observed in patients with ZEB2‐positive tumours when compared to ZEB2‐negative ones (Fig 1C). These results demonstrated for the first time that ZEB2 immunopositivity in colorectal tumours is an indicator of poor response to adjuvant chemotherapy, as highlighted by reduced patient survival and increased recurrence. Association of clinicopathological variables with ZEB2 immunopositivity and patient demographics is listed in Tables S1‐S3 in compliance with international REMARK biomarker reporting standards [25]. Multivariable Cox regression analysis highlighted ZEB2 as an independent prognostic marker of OS (HR 3.13, 95% CI 1.59–6.16, *P* = 0.001) and DFS (HR 3.12, 95% CI 1.53–6.65, *P* = 0.002; Tables S4,S5) in patients after FOLFOX chemotherapy.

### 3.2 ZEB2 expression is retained in a subpopulation of cells in CRC liver metastases

ZEB proteins and members of the miR‐200 family form a double‐negative feedback loop implicated in the control of EMT and mesenchymal–epithelial transition (MET) balance, and *ZEB1* and *ZEB2* are often coregulated through this mechanism [26]. Analysis of *ZEB1* gene transcription demonstrated that metastatic lesions express less *ZEB1* than corresponding primary tumours, but more of miR‐200 [27] suggesting this negative feedback loop is effective in the MET process at secondary CRC. However, there are many examples of complex mutual regulation of EMT‐TFs, and recent evidence suggests that expression of ZEB1 and ZEB2 protein expression can be controlled differently at translational and post‐translational levels [28, 29]. In addition, the abundance and specificity of mir‐200 binding sites at the 3′‐UTR of *ZEB1* and *ZEB2* mRNA are different [30]. As (a) adjuvant chemotherapy is intended to kill cancer cells at secondary sites and (b) ZEB2 protein expression in secondary CRC has never been studied, we analysed 30 paired samples from patients who underwent surgical resection of primary CRC tumours and synchronous/metachronous liver metastases. Clinicopathological variables of this cohort are presented in Table S6. Nuclear ZEB2 was observed in 87% (26/30) of the primary CRC tumours and 83% (25/30) of the paired liver metastases (Fig. S1). More than 96% (25/26) of ZEB2‐positive primary tumours also stained positive for ZEB2 in their corresponding liver metastases. No ZEB2‐negative primary tumour exhibited ZEB2 in the recurrence. Taken together, these data suggest that ZEB2 is expressed in both primary and secondary CRC and patients with ZEB2 expression have poorer prognosis and limited response to chemotherapy.

### 3.3 ZEB2 promotes resistance to the DNA damaging agents used in the FOLFOX regimen

**Fig. 2:**
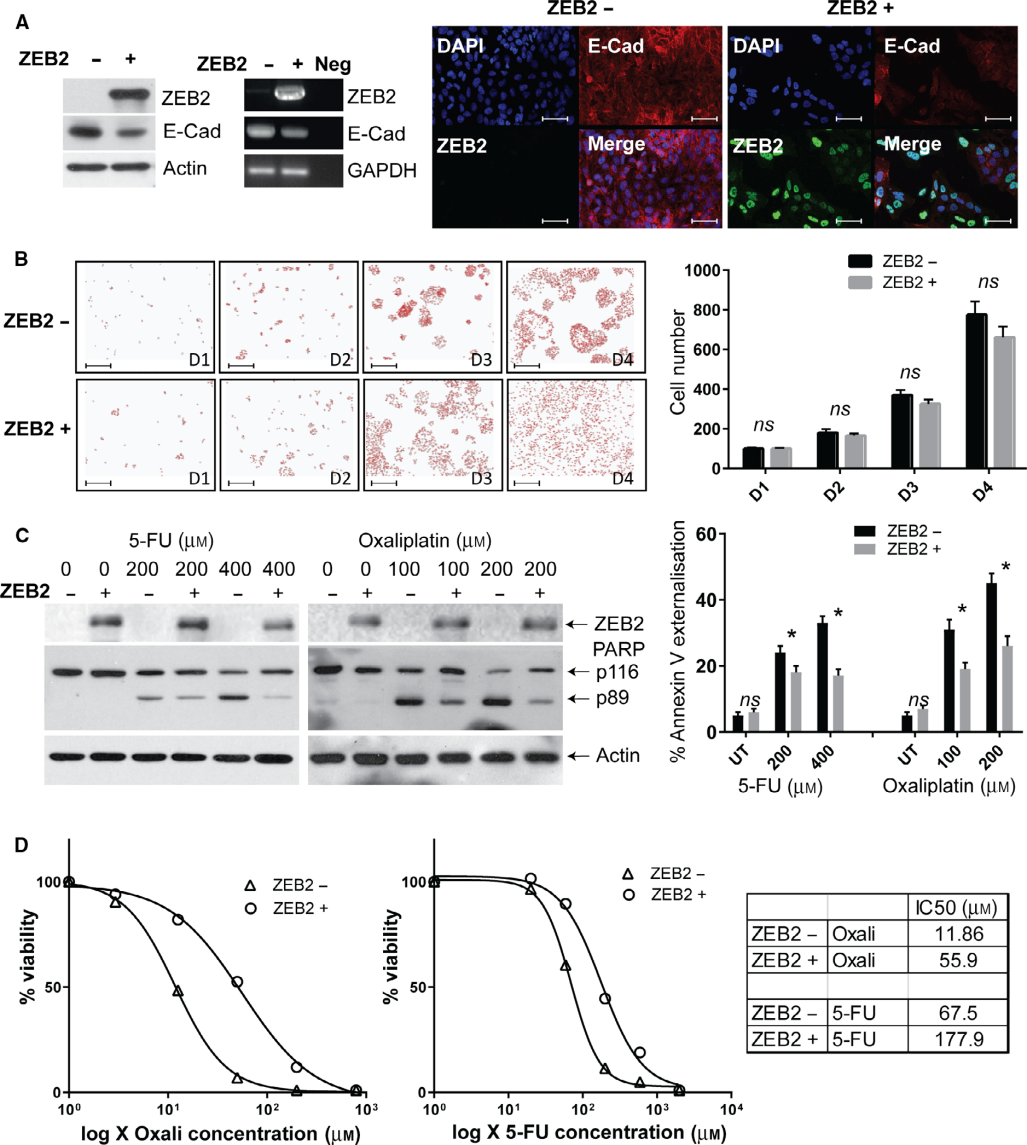
ZEB2 induces chemoresistance in CRC cells. (A) ZEB2 induction in DLD1‐ZEB2 cells by DOX results in the partial decrease in E‐cadherin levels as shown by immunoblotting (left panel), RT‐PCR (central panel) and immunofluorescence (right panel). The scale bars are indicating 50 μm. (B) ZEB2 induces cell scattering, but does not create a significant decrease in cell proliferation until day 4. Cells were cultured in the presence or absence of DOX, fixed with acetone/methanol and stained with DAPI to identify nuclei. Pictures were taken using 4× objective (left panel), and cell numbers (nuclei) were quantified using imagej software. Graph represents means (± SD) of an experiment performed in triplicate and statistical analysis performed using Student’s *t*‐test. The scale bars are indicating 100 μm. (C) DLD1‐ZEB2 cells became resistant to 5‐FU‐ and oxaliplatin‐induced cell death upon ZEB2 induction. Cells were maintained with or without DOX for 72 h and then treated with the indicated concentrations of drugs for 24 h, or treated with PBS. Western blot shows induction of ZEB2 and accumulation of cleaved PARP in treated cells. The extent of apoptosis was measured by Annexin V/PI staining and presented as graph (right panel). Graph represents means (±SDs) of three independent experiments and statistical analysis performed using Student’s *t*‐test. (D) IC50 curves indicate ZEB2 expressing DLD‐1 cells are resistant to 5‐FU or oxaliplatin induced growth inhibition. The graphs were presented on the left and numerical IC50 values were presented on the right as a table.

Cells undergoing EMT often acquire resistance to apoptotic stimuli [6]. As ZEB2 immunopositivity correlated with decreased OS and DFS observed in patients receiving FOLFOX (Fig. 1), we hypothesised that ZEB2 may be responsible for resistance of CRC cells to components of this regimen. Therefore, we investigated the impact of ZEB2 on chemoresistance to cytotoxic agents used in FOLFOX regimen (5‐FU and oxaliplatin) using a previously established CRC cell line with DOX‐inducible expression of ZEB2 (DLD1‐ZEB2 cells) [21]. As expected, treatment with DOX resulted in nuclear expression of ZEB2, cell scattering, downregulation of E‐cadherin (Fig. 2A) but no upregulation in vimentin (data not shown) suggestive of a partial EMT. We also did not observe a significant effect on proliferation but a trend of cell cycle slowing till day 4 of induction was evident (Fig. 2B). To analyse its antiapoptotic function, ZEB2 was induced for 3 days, and cells were subsequently treated with different concentrations of oxaliplatin or 5‐FU. ZEB2 protected DLD1 cells from apoptosis induced by either drug at different concentrations, as assessed by PARP cleavage and quantified by Annexin V/PI assay (Fig. 2C). Viability assays of ZEB2‐inducible DLD cells treated with oxaliplatin or 5‐FU support our previous findings that ZEB2‐induced EMT renders DLD cells chemoresistant (Fig. 2D). These results suggest that ZEB2‐expressing CRC cells acquire resistance to chemotherapeutic agents commonly used in CRC treatment and may explain the observed reduced survival in patients with ZEB2‐positive tumours who received FOLFOX chemotherapy despite inducing a partial EMT.

Enhanced drug efflux is a recognised mechanism of chemoresistance, and some EMT‐TFs are reported to induce multidrug resistance against chemotherapeutic agents such as doxorubicin, oxaliplatin and 5‐FU by upregulating ABC transporters [16, 31]. Although not used in the treatment of CRC, we used doxorubicin to investigate whether ZEB2 promotes apoptosis resistance by drug exclusion due to its intrinsic fluorescence properties and the fact that drug efflux mechanisms of doxorubicin oxaliplatin are similar [32]. ZEB2‐expressing CRC cells also showed resistance to doxorubicin‐induced apoptosis (Fig. S2A). Drug efflux capacity quantified by doxorubicin fluorescence, measured 4 h after an initial 1‐h exposure, revealed no significant effect of ZEB2 on intracellular (retained) doxorubicin (Fig. S2B,C) suggesting that enhanced drug efflux is unlikely to be the mechanism responsible for chemoresistance in DLD1‐ZEB2 cells.

### 3.4 Genes implicated in Nucleotide Excision Repair pathway are transcriptional targets of ZEB2

**Fig. 3:**
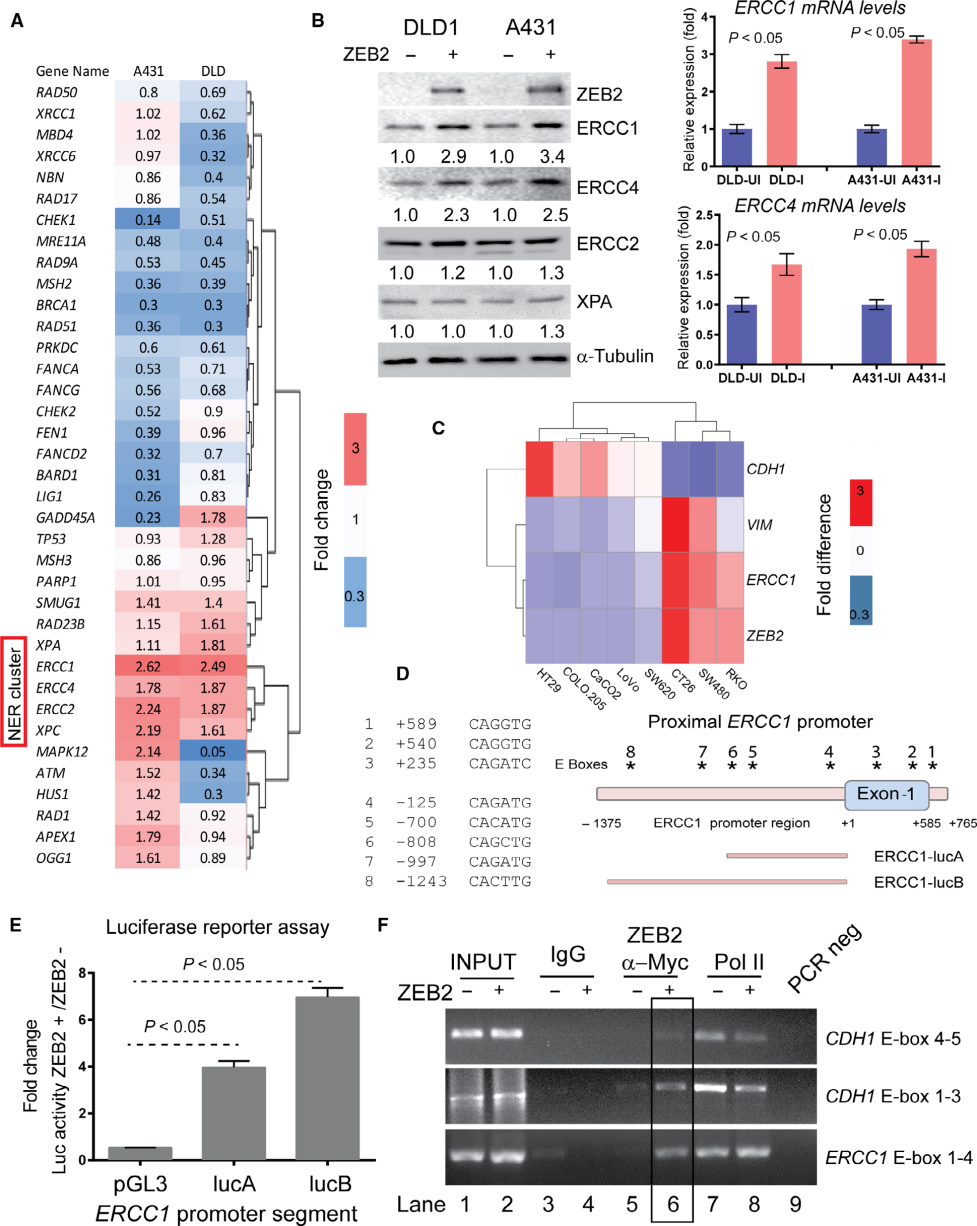
ZEB2 directly regulates ERCC1 expression. (A) ZEB2 activates transcription of genes implicated in NER pathway in two cell lines, DLD1 and A431. The heat map shows genes with significant up‐ or downregulation in response to the induction of ZEB2 (*P* < 0.05, Student’s *t*‐test). Note that genes belonging to the NER cluster are induced in both cell lines. (B) Validation of ZEB2‐induced gene expression. Left panel: Protein abundance of NER proteins, ERCC1, ERCC2, ERCC4 and XPA was assessed in both A431‐ and DLD‐ZEB2 cells. Band intensities were quantified against tubulin. Right panel: ZEB2 increased expression of ERCC1 and ERCC4 was assessed by qPCR. (*n* = 3 for all genes) (C) The expression of *CDH1*, *Vimentin*, *ERCC1* and *ZEB2* was assessed by qRT‐PCR in a panel of CRC cell lines and results presented as heat map.(*n* = 3 for all genes) (D) Schematic representation of potential ZEB2‐binding sites (E‐boxes, CANNTG) localised in the vicinity of the *ERCC1* transcription start site (on the right), and their sequences (on the left). The portions of *ERCC1* promoter cloned to assess luciferase activity upon ZEB2 induction were indicated below the graph (E) Activity of reporters containing fragments A and B of the *ERCC1* promoter (Fig. 3C) in DLD1 cells in the presence or absence of ZEB2. The reporter activity in ZEB2‐expressing cells was normalised to that of the reporters in DLD1 cells in the absence of ZEB2. Data represent mean ±SD of three independent experiments (*n* = 3). (F) ZEB2 binds to regulatory regions of *ERCC1*. Exogenous ZEB2 was immuno‐precipitated by an antitag (c‐MYC) antibody; IgG and anti‐RNA Pol II antibodies were used as controls. ZEB2 enrichment was detected at both *ERCC1* and *CDH1* (positive control for ZEB2 binding) regulatory regions (lane 6 highlighted by a box as compared to lane 5). *CDH1* E‐boxes 1‐3 and 4‐5 are located at the positions −859, −575, −399, +39 and +89, respectively, relative to the *CDH1* transcription start site (ENSEMBL ID: CDH1‐201, ENST00000261769.9) and detailed in Fig. S4.

We next explored the mechanism by which ZEB2‐expressing cells acquire resistance to chemotherapy‐induced apoptosis. To identify relevant ZEB2 transcriptional targets, we employed a qPCR array for genes implicated in DNA damage response/repair by using two ZEB2‐inducible cell lines (A431‐ZEB2 and DLD1‐ZEB2) [21]. Hierarchical clustering of gene expression profiles revealed that ZEB2 consistently activated genes implicated in NER pathway. All NER genes represented on the array including *ERCC1*, *ERCC2*, *ERCC4 (XPF)*, *XPA* and *XPC,* were upregulated by ZEB2 in both cell lines, (Fig. 3A). Consequently, we next investigated the expression of NER cluster genes during ZEB2‐induced EMT. Among 4 proteins analysed, ERCC1 and ERCC4 demonstrated the biggest induction both at RNA and protein levels upon ZEB2 expression (Fig. 3B). NER is an evolutionary conserved DNA repair mechanism, responsible for removal of DNA adducts generated by UV, UV‐mimetic compounds (e.g. mitomycin C) or platinum‐based chemotherapeutic agents [33]. Due to the requirement of excising the damaged part of DNA during NER and/or creating a free 3’ DNA end for repairing/filling of damaged DNA sections, the NER proteins ERCC1/ERCC4, constituting a 5‐exonuclease function, also gained importance in double‐strand break (DSB) repair and interstrand DNA crosslink repair mechanisms [34]. Therefore, ERCC1 and ERCC4 are also critical in the DNA damage response that can be caused by a variety of insults such as UV, radiation, DNA crosslinking agents, topoisomerase inhibition and toxic metabolite intermediates. To investigate the impact of ERCC1‐ERCC4 proteins to oxaliplatin resistance as individuals or as a complex, we performed knockdown experiments (Fig. S3A). Our results suggest that siRNA‐mediated downregulation of *ERCC1* or *ERCC1* and *ERCC4* expression had a similar and bigger effect as compared to *ERCC4* knockdown alone (Fig S3B). These results confirm reports in existing data suggesting deficiency of any component of NER machinery may render cells sensitive to DNA crosslinking agents; however, *ERCC1* heterozygosity/loss produced the most prominent DNA repair‐deficient phenotype [35]. Therefore, efficient removal of oxaliplatin‐induced DNA crosslinks via upregulation of ERCC1 and ERCC4 may therefore represent a survival strategy that could be adopted by ZEB2‐expressing CRC cells to evade cell death. Importantly, the formation of ERCC1‐ERCC4 complex has been shown to stabilise the individual proteins; therefore, the concominant increase of these proteins is not a surprise [36]. To test whether the increase in *ERCC1* expression we observed (e.g. ~ 3‐fold in RNA and protein levels) is functionally relevant, we investigated expression levels of *ERCC1* in normal human tissues. *ERCC1* is ubiquitously expressed in all 53 normal tissues (mean expression = 23 ± 7.70 units) of the GTEx cohort of Expression Atlas database [37]. Skin is the only tissue constantly exposed to sunlight, a major source of DNA damage repaired via NER. *ERCC1* was expressed in the skin (31.30 ± 0.33 units) at a level that is twofold (*P* = 0.03) higher than in the tissues of internal organs of the digestive system, including large intestine (16.10 ± 4.00 units; Fig. S3C). These results suggest that ERCC1 expression is ubiquitous, and a twofold increase in its expression levels may be important for its biological function. We, then, tested whether *ERCC1* expression correlated with levels of *ZEB2* and EMT markers in CRC cell lines. Quantitative PCR analysis of 8 CRC cell lines showed that *ERCC1* expression is associated with an absence of *CDH1* and high expression of *ZEB2* and *Vimentin* (Fig. 3C). In the CCLE cohort of the Expression Atlas database, where 41 CRC cell lines are present [37], *ERCC1* expression was highest in cell lines with low *CDH1* and high *ZEB2* (Fig. S3D). These observations suggest ERCC1 overexpression is a component of the ZEB2‐induced EMT programme, and may contribute to oxaliplatin resistance of mesenchymal CRC cells.

We next evaluated whether *ERCC1* is a direct transcriptional target of ZEB2. We identified eight putative E‐box elements (CANNTG) in the 2 kb regulatory region encompassing the promoter and the first exon of the *ERCC1* gene (Fig. 3D). We therefore cloned *ERCC1* promoter into PGL3‐basic vector as two DNA fragments (ERCC1‐luc‐A and ERCC1‐luc‐B, Fig. 3D). ZEB2 induction mediated a significant increase in luciferase signal in cells transfected with the reporters harbouring promoter fragments A or B, containing E‐boxes 4‐8 (Fig. 3D,E). Luciferase activity was reduced as the number of E‐boxes in the cloned fragment decreased suggesting that ZEB2 binding to E‐boxes contributes to the increased gene expression. We next conducted chromatin immunoprecipitation (ChIP) to analyse ZEB2 binding to E‐boxes 1‐4 and 5‐7 of *ERCC1* (described in Fig. 3D) and *CDH1* (positive control, described in Fig. S4). We detected interactions of ZEB2 with the regulatory regions of both the *ERCC1* and *CDH1* (Figs 3F and S5). The functional effects (transcriptional upregulation or downregulation) of this binding were demonstrated by alterations in RNA polymerase II occupancy which marks actively transcribed segments of DNA (Figs 3F and S5). These results demonstrate that ZEB2 directly binds to the regulatory regions within the *ERCC1* locus and upregulates its expression.

### 3.5 ERCC1‐overexpressing CRC cells exhibit less DNA damage, attenuated DNA damage response, reduced apoptosis and enhanced resistance to oxaliplatin *in vitro*

**Fig. 4:**
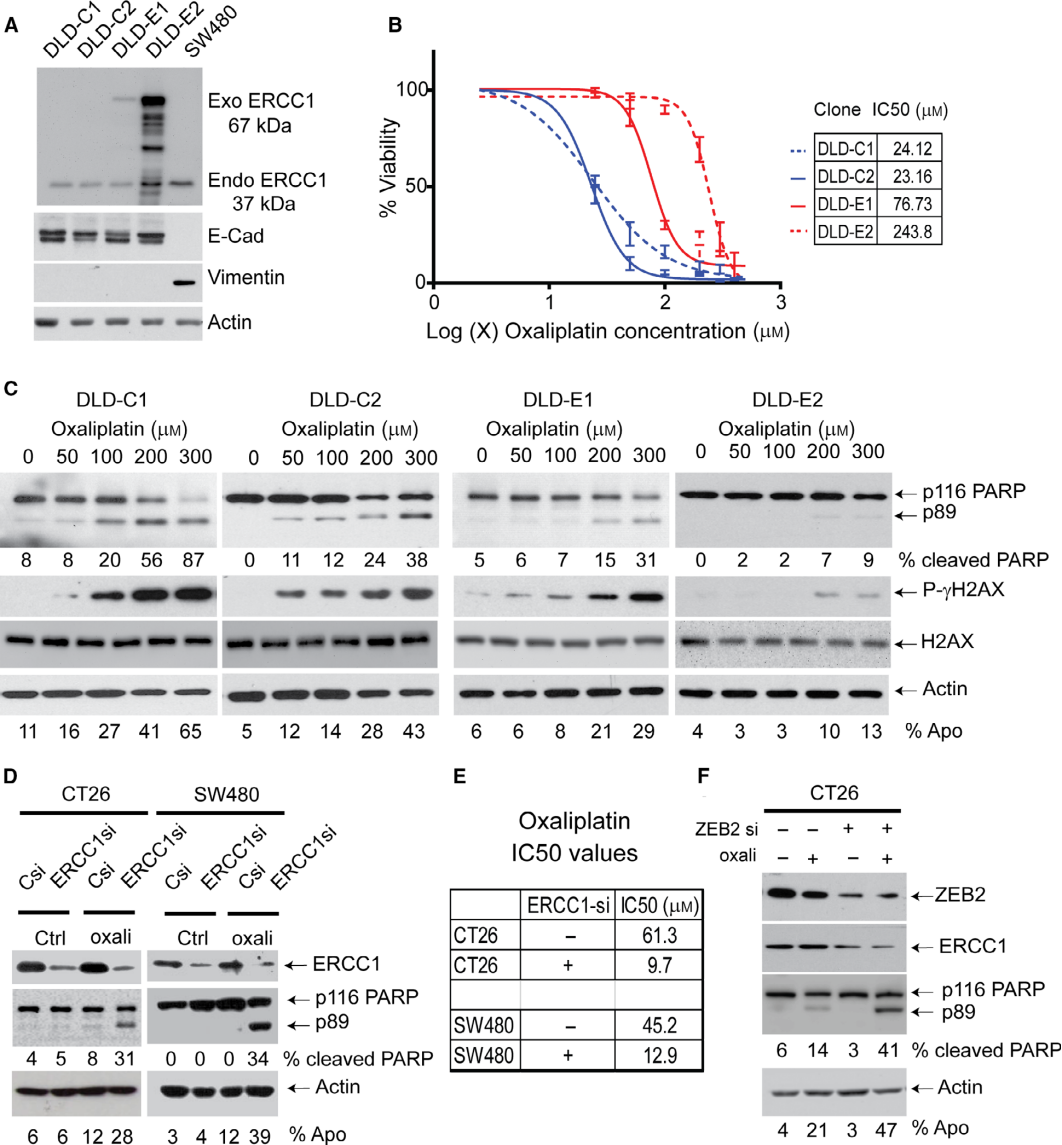
ERCC1 induces oxaliplatin resistance. All experiments in this figure have been repeated at least three times (*n* = 3) and a representative figure was presented (A) Western blot analysis of the ERCC1 expression in DLD1‐derived cell lines overexpressing ERCC1 (DLD1‐E1 and ‐E2), and control clones (DLD1‐C1 and DLD1‐C2). Note that exogenous (exo) ERCC1 (67 kDa) was also detected by ERCC1 antibody and clone E1 expressed ERCC1 at a similar level to the mesenchymal CRC cell SW480 (last lane). None of the clones, either control or overexpressing ERCC1, exhibited mesenchymal properties which can occur due to neomycin selection. (B) Cell viability assay for control and ERCC1‐overexpressing clones demonstrates that ERCC1 expression level is the determinant of *IC50* values (table) in cells treated with oxaliplatin. Data represent mean ± SD of four independent experiments (C) ERCC1 clones were treated with indicated concentrations of oxaliplatin. The extent of PARP cleavage (p89‐PARP) and γ‐phosphorylated H2AX levels was assessed by immunoblotting to determine the magnitude of apoptosis and DNA damage response pathway activation. DLD1‐EC1 and DLD1‐EC2 cells showed reduced pro‐apoptotic signalling as compared to the control clones, DLD1‐C1 and DLD1‐C2. The cleavage of PARP and presence of Annexin V positivity (% Apo) were presented as quantitative measures of apoptosis at the bottom of blots. (D) *ERCC1* has been knocked down in mesenchymal CRC cell lines CT26 and SW480. These cells were treated with 100 mm oxaliplatin for 24 h. Control cells demonstrated minimal apoptotic response, whereas ERCC1 depletion (ERCC1si) induced cell death, as assessed by PARP cleavage (formation of p89‐PARP) and Annexin V externalisation. (E) ERCC1 knockdown also significantly reduced oxaliplatin IC50 values of both CT26 and SW480 cells. (F) *ZEB2* knockdown in CT26 cells using siRNA. The abundance of ERCC1 protein, in the absence or presence of oxaliplatin treatment, was assessed by western blotting. Oxaliplatin‐induced apoptosis was increased as a result of decreased ERCC1 level and assessed by PARP cleavage (presence of p89‐PARP) and Annexin V externalisation (presented as % Apo).

We next studied whether ERCC1 is the main determinant of oxaliplatin resistance in ZEB2‐expressing CRC cells. Parental (wt) DLD1 cells were transfected with expression vectors harbouring mCherry‐ERCC1 or mCherry alone (control) to generate stable cells lines. Two clones expressing different levels of ERCC1 were selected for further analysis. Although all clones displayed an epithelial phenotype (Fig. 4A), ERCC1‐overexpressing (DLD1‐E1 and DLD1‐E2) cells exhibited threefold to tenfold increased resistance to oxaliplatin treatment compared to controls (DLD1‐C1 and DLD1‐C2) as determined by cell viability assays (Fig 4B). These cell lines also showed enhanced resistance to apoptosis (less Annexin V externalisation and reduced PARP cleavage) and attenuated γH2AX phosphorylation (a marker of DNA damage) (Fig. 4C). To make sure the attained apoptosis resistance is due to ERCC1 overexpression but not as a result of clonal selection, we reduced ERCC1 expression in DLD‐C1 and DLD‐E1 cells by siRNA and treated these cells with oxaliplatin. Our results suggest a marked increase in annexin V externalisation and PARP cleavage, as well as a decreased IC50, indicative of apoptosis, when ERCC1 protein expression is reduced (Fig S6A,B). Further, to investigate whether mesenchymal CRC cell lines are less sensitive to oxaliplatin due to high endogenous ERCC1 expression, we depleted *ERCC1* in CT26 and SW480 cells, in which both ZEB2 and ERCC1 expression were also high (Fig. 3C). ERCC1 siRNA‐mediated reduction in ERCC1 protein expression resulted in sensitisation of both cell lines to oxaliplatin‐induced apoptosis (Fig. 4D). These cells also displayed decreased viability (lower *IC50* values) as assessed by crystal violet assay (Fig. 4E). To further assess the association between ZEB2 and ERRC1 expression, we knocked down *ZEB2* in CT26 cells and investigated ERCC1 expression and apoptotic response to oxaliplatin. We observed acquired sensitivity to oxaliplatin along with ERCC1 downregulation (Fig. 4F). Our results suggest that high protein abundance of ERCC1 enhances increased viability and resistance to apoptosis upon oxaliplatin exposure. However, we cannot exclude the contribution of other NER components, including ERCC4, towards ZEB2‐induced chemoresistance.

### 3.6 Increased ERCC1 expression promotes enhanced DNA repair kinetics

**Fig. 5:**
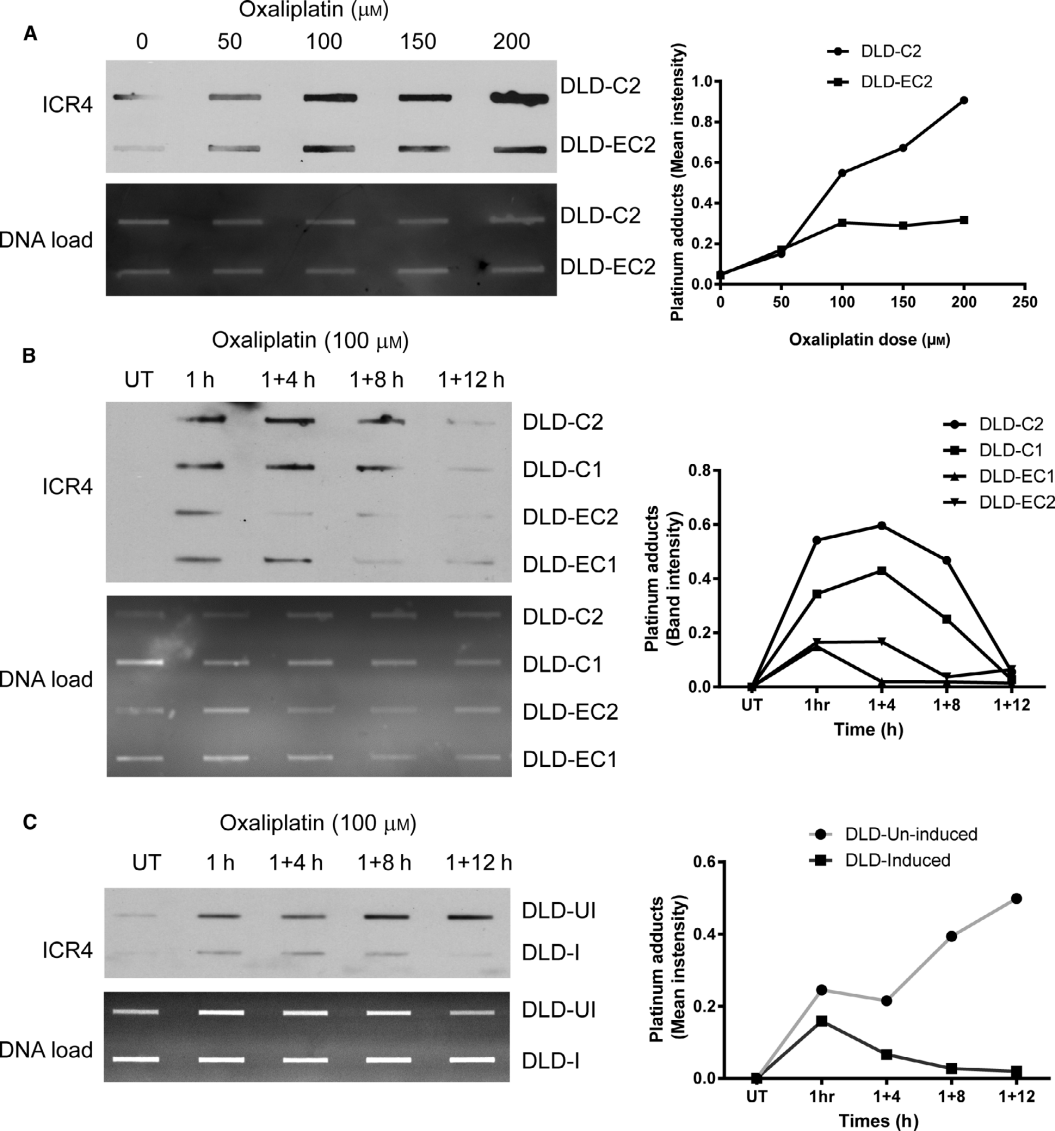
ERCC1 overexpression augments clearance of platinum‐DNA crosslinks. All experiments in this figure have been repeated at least three times (*n* = 3) and a representative figure (left) and quantification (right) are presented. Untreated cells (UT). (A) DLD1‐C2 and DLD1‐E2 cells were treated with indicated concentrations of oxaliplatin for 4 h. Genomic DNA was isolated and equal amount (1 μg) from each sample was transferred to nitrocellulose membrane. Platinum‐DNA adduct antibody (ICR4) was used to assess the abundance of oxaliplatin‐induced DNA damage (upper panel). Lower panel shows total DNA load. Graph (means ± SD of three independent experiments) shows quantification of DNA adducts detected by the ICR4 antibody and normalised to the total DNA in a sample. (B) Control and ERCC1‐overexpressing DLD1 clones were treated with 100 μm oxaliplatin for 1 h, washed and assessed for DNA repair capacity at different time points as explained in Fig. 5A. Note that DLD1‐E1 and DLD1‐E2 cells cleared oxaliplatin‐induced DNA crosslinks quicker than controls indicating faster DNA repair. (C) DLD1‐ZEB2 cells were cultured in the absence or presence of DOX for 3 days, incubated with 100 μm oxaliplatin, washed and assessed for platinum adduct clearance. Induced cells (ZEB2+) displayed less damage and quicker recovery as compared to ZEB2‐negative cells.

Next, we investigated whether enhanced expression of ERCC1 is sufficient for the stimulation of NER in CRC cells. The platinum adduct antibody (ICR4) was used to detect DNA damage and to quantify the extent of DNA repair after oxaliplatin treatment. ERCC1‐overexpressing cells demonstrated less DNA damage at all doses tested compared to controls (Fig. 5A). Furthermore, DLD1‐E1 and DLD1‐E2 cells exhibited enhanced DNA repair kinetics. A 1‐h oxaliplatin treatment and 12 h recovery revealed that the kinetics of DNA repair correlated with ERCC1 protein abundance in these clones (Fig. 5B). A similar pattern of enhanced DNA repair kinetics was also observed in ZEB2‐expressing DLD1 cells compared to ZEB2‐negative counterparts (Fig. 5C). Interestingly, the initial DNA damage (at 1 h) seems less in ZEB2‐positive cells as compared to ZEB2‐negative ones. This observation can be attributed to the fact that ZEB2 expression has already induced ERCC1; therefore, these cells were able to repair some of the damage during the course of the treatment (1 h). Alternatively, ZEB2‐induced EMT programme could be contributing to other aspects of DNA damage recognition/repair pathways through coregulated factors. Nevertheless, our findings confirm that ERCC1 expression is one of the key factors in NER pathway in CRC cells, and ZEB2‐induced ERCC1 enhances DNA repair capacity.

### 3.7 Enhanced ERCC1 expression limits tumour response to oxaliplatin treatment *in vivo*

**Fig. 6:**
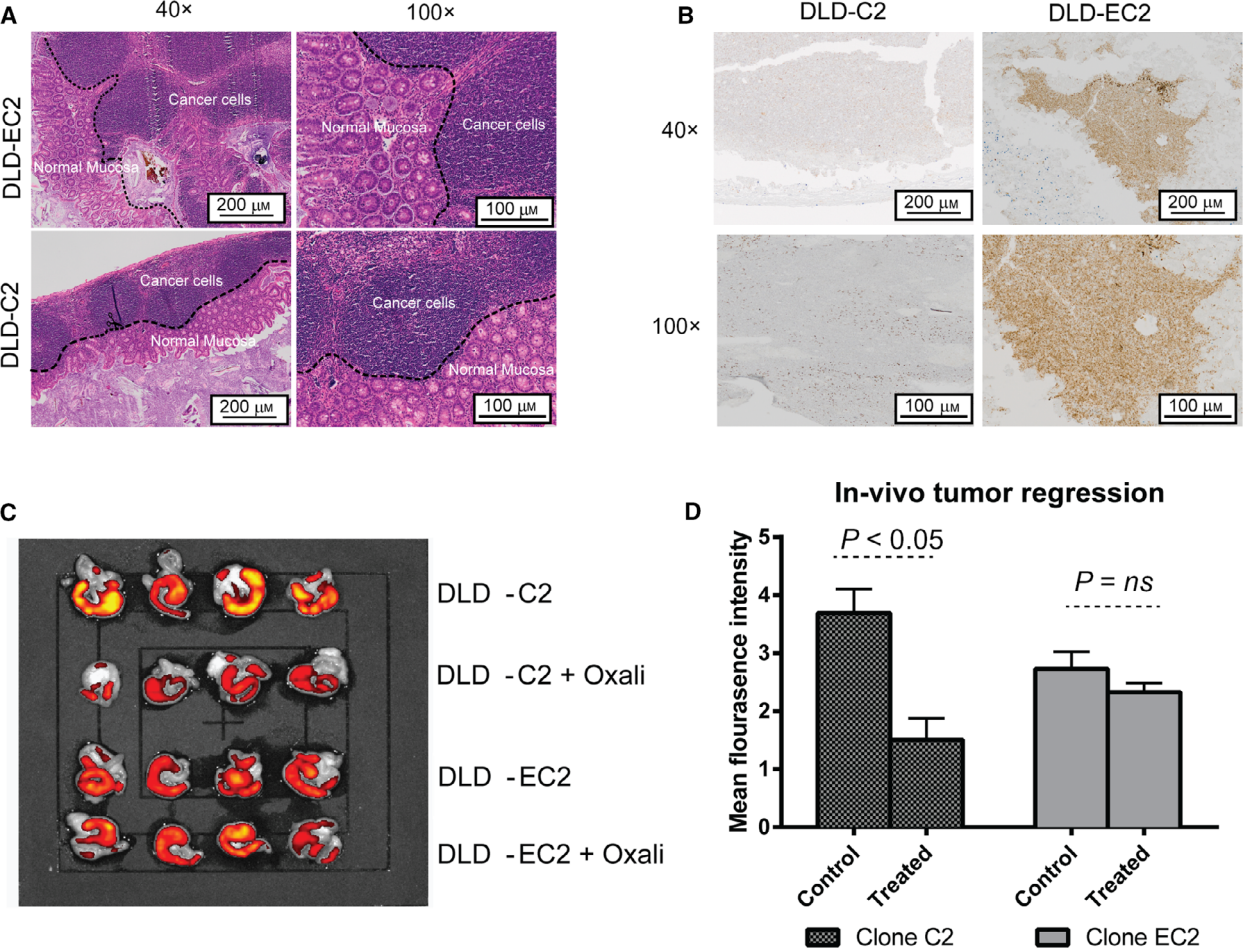
Exogenous ERCC1 reduces tumour response to oxaliplatin in an orthotopic mouse model of CRC. (A) DLD1‐E2 and DLD1‐C2 cells were implanted in the caecum of immunocompromised mice. After 4 weeks, animals were randomly allocated to treatment (oxaliplatin) or control (PBS) groups. Oxaliplatin (5 mg·kg^−1^·week^−1^) and PBS were administered weekly by intraperitoneal injection. Noninvasive imaging confirmed the presence of primary tumours in all mice. At week 9 post‐implantation, mice were culled and caecum, liver, lungs and spleens were harvested and subjected to further imaging and histopathology. Both cell lines produced tumours of similar size, which exhibited a phenotype of differentiated CRC. (B) IHC analyses of ERCC1 expression in tumours formed by DLD1‐E2 and DLD1‐C2 cells prove retained ERCC1 expression in clone DLD‐E2. Please note that ERCC1 IHC has been optimised to detect exogenous ERCC1 as both DLD‐E2 and DLD‐C2 express similar amounts of endogenous ERCC1, as described in Fig. 4A. (C‐D) Imaging and quantification of primary DLD1‐C2 and DLD1‐E2 tumours with or without oxaliplatin treatment. Excised caecums were imaged. Data represent mean ± SD of three samples and significance (*P* value) was assessed using Student’s *t*‐test considering equal sample size but assuming equal or unequal variance (Welch’s test). Please note that oxaliplatin administration significantly reduced fluorescence signals in DLD1‐C2 but not in DLD1‐E2 tumours as compared to PBS treated control. The starting fluorescence of DLD‐E2 was less than DLD‐C2, likely because the mCherry‐ERCC1 fusion protein emits less light than wild‐type mCherry.

We next aimed to test whether enhanced levels of ERCC1 impact on chemotherapy resistance *in vivo*. We analysed (DLD1‐E2) and control (DLD1‐C2) cells in an orthotopic murine model based on direct intracaecal implantation of tumour cells. Mice were allowed to recover from surgery and allocated randomly to PBS (control) or oxaliplatin treatment groups. Oxaliplatin and PBS were administered intraperitoneally at weekly intervals once animals register emitting fluorescence (from mCherry) as assessed by *in vivo* imaging. At end point, tumours were excised allowing quantitative and qualitative analysis. Tumour presence and ERCC1 expression in tumours were confirmed by histopathological analysis by pathologists blinded to treatment received (Fig. 6A,B). Primary tumours expressing low/no ERCC1 (DLD‐C2) exhibited a significant reduction in fluorescence signal and tumour weight upon oxaliplatin treatment indicating tumour shrinkage (Figs 6C,D and S7). ERCC1‐overexpressing primary tumours, however, did not show any significant response to oxaliplatin (Fig. 6C,D and S7). Distant metastases were not detected in any animal in the study time frame (assessed by fluorescence imaging and histopathological analysis upon necropsy; data not shown). These findings suggest that ERCC1 overexpression contributes to oxaliplatin resistance in CRC *in vivo*, and highlight why ZEB2‐expressing CRC tumours may demonstrate limited response to FOLFOX therapy.

## 4 Discussion

Metastasis and therapy resistance are major causes of cancer‐associated mortality [1]. A growing body of data suggests EMT signature is associated with poor oncological outcomes in CRC [38]. In this study, we demonstrate that nuclear ZEB2 expression predicts early recurrence and reduced survival in patients that received adjuvant FOLFOX chemotherapy after a surgical resection for primary CRC. ZEB2 enhanced resistance of CRC cells to oxaliplatin‐induced DNA damage, by increasing NER capacity. Enhanced DNA repair resulted in reduced pro‐apoptotic signalling and treatment resistance both *in vitro* and *in vivo*.

Chemotherapeutic regimens encompassing use of conventional DNA damaging agents (FOLFOX, FOLFIRI) continue to represent the main treatment option in patients with CRC [39]. Although the association between chemo/radioresistance and EMT has been documented previously, the mechanistic details remain poorly understood and are likely to be multifactorial [15, 40]. Clinical studies suggest CRC patients with tumours belonging to the mesenchymal molecular subtype exhibit poor response to conventional adjuvant chemotherapy [5]. We previously reported ZEB2 expression promotes resistance to DNA damage‐induced apoptosis due to attenuated ATM/ATR activation [15]. This finding may have different explanations: ZEB2 either compromises recognition of the damaged DNA or enhances repair capacity. Our results suggest that faster and more efficient repair of oxaliplatin‐induced damage is the main contributing factor to chemoresistance. A central role for ZEB1 in promoting resistance to ionising radiation by enhancing homologous recombination has also been demonstrated recently [41]. Hence, ZEB‐mediated activation of DNA repair pathways emerges as a viable cellular strategy to gain chemo/radioresistance.

The majority of DNA damage inflicted by platinum‐based chemotherapeutic agents are intrastrand G‐G dimers [42]. These DNA adducts distort DNA helix, inhibit replication and transcription to drive cells into apoptosis [35]. Of several DNA repair systems in eukaryotic cells, NER is credited with playing a central role in removal of platinum induced DNA adducts [35]. The process of intrastrand crosslink removal involves, DNA damage recognition, unwinding, adduct excision by endonucleases, DNA re‐synthesis and ligation. The key feature NER is the introduction of incisions by XPG (at 3′) and ERCC1‐XPF complex (at 5′) into the damaged DNA strand on either side of the adduct, resulting in the excision and removal of a single strand DNA fragment [43]. In contrast, interstrand crosslinks are converted into a DSB during DNA replication where the ERCC1‐XPF complex plays a key role in removal of nonhomologous 3′ single‐stranded flaps, which is subsequently repaired by homologous recombination [34]. In this study, we demonstrate ZEB2‐induced ERCC1 expression results in faster clearance of platinum adducts, possibly of both intra‐ and interstrand types, (Fig. 5) thus enhancing chemoresistance (Fig. 6).

Among all NER proteins, ERCC1 stands out as it is also involved in Fanconi Anaemia pathway, interstrand crosslink repair and DSB repair [44]. Hypersensitivity of ERCC1 mutants to DNA crosslinking agents, when compared to other NER proteins, is also well accepted despite only functioning as the noncatalytic component of 5′‐exonuclease NER complex comprised of ERCC4 and ERCC1 [35]. In CRC cell lines, mRNA levels of *ERCC1* directly correlated with enhanced repair capacity, while small interfering RNA‐mediated knockdown increased sensitivity, as shown in this article and by others [45, 46]. Bladder and lung cancer cell lines that exhibit resistance to platinum derivatives were shown to possess enhanced NER activity [47, 48]. The hypersensitivity of testicular cancer to platinum‐based chemotherapy is associated with low abundance of ERCC1 and impaired DNA repair capacity [49]. In early clinical trials, high ERCC1 expression has also been associated with cisplatin resistance in ovarian and non‐small‐cell lung cancers [50, 51], however, later refuted. To date, the cellular mechanisms controlling ERCC1 expression and promoting intrinsic platinum resistance in CRC have remained elusive. Here, we demonstrate, for the first time, that the activation of EMT by ZEB2 is instrumental for ERCC1 overexpression, enhanced NER capacity and oxaliplatin resistance.

There has been significant attention on the clinical use of ERCC1 as a predictive biomarker of response to platinum‐based therapies; however, IHC, RNA expression and ERCC1 polymorphisms have been trialled with mixed results [52]. The FOCUS trial (*n* = 1197) utilised IHC to associate ERCC1 protein expression to predict clinical response to platinum treatment. ERCC1 expression did not predict response to FOLFOX therapy [53]. Analysis of ERCC1 protein expression in clinical samples is complicated by the existence of four protein isoforms that differentially impact DNA repair. Of the recognised isoforms, only ERCC1‐202 (297aa) was associated with nucleotide excision and interstrand crosslink repair capacity [44]. An investigation of commercially available ERCC1 antibodies proved their inability to differentiate between the four isoforms, consequently, rendering specific detection of functionally relevant ERCC1 by IHC impossible [54]. Therefore, we did not attempt to stain our CRC cohorts with ERCC1 and associate its expression with ZEB2. Another important biasing feature of ERCC1 is its expression pattern. *ERCC1* is a ubiquitously expressed gene, and as shown in this article, its expression is increased threefold during EMT. Although ERCC1 upregulation impacted oxaliplatin response significantly, it has proven to be difficult to quantify accurately by IHC without a standardised reporting system.

A paucity of data relating to ZEB2 expression and its association with chemoresistance exists due to the absence of effective antibodies that have been meticulously validated. We overcame this hurdle by generating and validating our own SIP1/ZEB2 antibody and demonstrating specificity in western blotting and IHC [15, 20, 22]. Kahlert et al previously reported cytoplasmic expression of ZEB2 at the invasive front of primary CRC’s prognosticated for poor cancer specific survival [55]. However, the nuclear expression of ZEB2 and its prognostics value to differentiate response to chemotherapy were not addressed. In this study, we investigated and validated for the first time, the prognostic value of ZEB2 expression in a cohort CRC patients, who received adjuvant FOLFOX therapy after surgical resection of the primary tumour. Nuclear ZEB2 expression was associated with poor oncological outcomes in terms of both OS and DFS. We emphasised the importance of nuclear expression of ZEB2 in our scoring system and in our earlier publications [15, 20], as ZEB proteins are transcription factors executing their function mainly in the nucleus. It is also noteworthy that chemoresistance observed in cancer patients after neoadjuvant chemotherapy is intended to eliminate malignant cells at the secondary site. EMT‐inducing transcription factors are usually assessed from primary cancer and their expression associates with chemoresistance but how or why they contribute therapy resistance or even if they are expressed at the secondary site is not considered. Our study revealed ZEB2 expression is consistently retained at the metastatic site therefore making ZEB2 a functionally important molecule to consider for prognostication and therapy.

Due to the intrinsic complexity of scoring ERCC1 using IHC, we propose ZEB2 is a promising candidate biomarker, predicting FOLFOX resistance in patients with primary CRC. Unlike ERCC1, nuclear ZEB2 protein is very low/undetectable in normal colonic epithelium, but exclusively detected in CRC cells, which simplifies scoring and its application as a clinical tool. Another important feature of ZEB2, as shown in this article, is its capacity to induce resistance to both components of the FOLFOX regimen. The molecular mechanism driving 5‐FU resistance in CRC was highlighted as upregulation of thymidylate synthase [56]; however, the contribution of ZEB2 to this process should be investigated in a separate study.

## 5 Conclusion

Here, we show that ZEB2 expression is associated with multiple aspects (metastasis and chemoresistance) of CRC progression, making it a valuable biomarker to prognosticate disease trajectory. Further validation and progression to a prospective clinical trial will aid the application of ZEB2 immuno‐expression as a useful clinical tool in the near future.

## Conflict of interest

The authors declare no conflict of interest.

## Author contributions

RS, AES, ET, TY and AM developed the concept; RS, NC, AP, JNP and AM established the CRC patient database and collected CRC material; KM, MSN, MAL and GJT performed IHC, scored IHC staining and performed histopathological analysis of human and mouse tissues; RS, HAS, MM, AP, SK, MC, SE and ME performed laboratory experiments; RS and AES performed statistical analysis and *in silico* experiments; GB, ET, AES and HAS created cell line models; and RS, BDP, AM, ET, TY and AES wrote the paper.

## Supporting information


**Table S1**. Clinicopathological parameters of patients in the pilot and validation study.
**Table S2**. Clinical and pathological parameters of patients in the pilot and validation study and their association with nuclear ZEB2 expression.
**Table S3**. Methodological detail of the biomarker study reported in accordance with REMARK guidelines.
**Table S4**. Multivariate analysis (Cox proportional hazard regression model) of prognostic parameters for overall survival in colorectal cancer patients who received adjuvant FOLFOX therapy.
**Table S5**. Multivariate analysis (Cox proportional hazard regression model) of prognostic parameters for disease‐free survival in colorectal cancer patients who received adjuvant FOLFOX therapy.
**Table S6**. Clinical and pathological parameters of patients with primary colorectal cancer and matched colorectal liver metastases.
**Table S7**. Primers used in the study.
**Fig. S1**. ZEB2 expression in CRC liver metastasis.
**Fig. S2**. ZEB2 has no effect on drug efflux in DLD1 cells.
**Fig. S3**. Contribution of ERCC1 and ERCC4 to oxaliplatin response.
**Fig. S4**. The promoter and regulatory region of *CDH1*.
**Fig. S5**. The enrichment of E‐boxes in the promoter of *CDH1* (E‐box 4‐5) and the E‐box cluster (E‐box 5‐7) of *ERCC1*.
**Fig. S6**. ERCC1 overexpression but not clonal selection is responsible for oxaliplatin resistance of DLD clones.
**Fig. S7**. Weight of tumours obtained from DLD‐C2 or DLD‐E2 cells, with or without oxaliplatin treatment.

## Data Availability

The data that support the findings of this study are available from the corresponding author (a.e.sayan@soton.ac.uk) upon reasonable request. Clinicopathological data related to patients used in this study are available in (Tables S1–S6) of this article.
