# Supplementary material for: The ZEB2‐dependent EMT transcriptional programme drives therapy resistance by activating nucleotide excision repair genes ERCC1 and ERCC4 in colorectal cancer
Source: Mol Oncol. 2021 May 1;15(8):2065–83. doi: 10.1002/1878-0261.12965 (PMC8333771; doi:10.1002/1878-0261.12965)
Supplement: Supplementary file 1 — Table S1. Clinicopathological parameters of patients in the pilot and validation study. Table S2. Clinical and pathological parameters of patients in the pilot and validation study and their association with nuclear ZEB2 expression. Table S3. Methodological detail of the biomarker study reported in accordance with REMARK guidelines. Table S4. Multivariate analysis (Cox proportional hazard regression model) of prognostic parameters for overall survival in colorectal cancer patients who received adjuvant FOLFOX therapy. Table S5. Multivariate analysis (Cox proportional hazard regression model) of prognostic parameters for disease‐free survival in colorectal cancer patients who received adjuvant FOLFOX therapy. Table S6. Clinical and pathological parameters of patients with primary colorectal cancer and matched colorectal liver metastases. Table S7. Primers used in the study. Fig. S1. ZEB2 expression in CRC liver metastasis. Fig. S2. ZEB2 has no effect on drug efflux in DLD1 cells. Fig. S3. Contribution of ERCC1 and ERCC4 to oxaliplatin response. Fig. S4. The promoter and regulatory region of CDH1. Fig. S5. The enrichment of E‐boxes in the promoter of CDH1 (E‐box 4‐5) and the E‐box cluster (E‐box 5‐7) of ERCC1. Fig. S6. ERCC1 overexpression but not clonal selection is responsible for oxaliplatin resistance of DLD clones. Fig. S7. Weight of tumours obtained from DLD‐C2 or DLD‐E2 cells, with or without oxaliplatin treatment. [file MOL2-15-2065-s001.pdf]

The ZEB2-dependent EMT transcriptional programme drives therapy resistance by activating nucleotide excision repair genes *ERCC1* and *ERCC4* in colorectal cancer.

Rahul Sreekumar, Hajir Al-Saihati, Muhammad Emaduddin, Karwan Moutasim, Massimiliano Mellone, Ashish Patel, Seval Kilic, Metin Cetin, Sule Erdemir, Marta Salgado Navio, Maria Antonette Lopez, Nathan Curtis, Tamer Yagci, John N. Primrose, Brendan D. Price, Geert Berx, Gareth J. Thomas, Eugene Tulchinsky, Alex Mirnezami, A. Emre Sayan.

Supplementary Material

1. Supplementary Tables

2. Supplementary Figures

3. References

Table 1: Clinico-pathological parameters of patients in the pilot and validation study.

	Pilot study		Validation study	
	**n**	**%**	**n**	**%**
**Age (Yrs)**				
<60	2	5.8	24	24.3
>60	32	94.6	75	75.8
**Sex**				
Male	16	47.0	53.5	53.5
Female	18	53.0	46.5	46.5
**Site of tumours**				
Right	15	44.1	34	34.3
Left	15	44.1	34	34.3
Rectum	4	22.8	30	30.3
Missing	0	0	1	1
**Differentiation**				
Well	0	0.0	3	3.0
Moderate-well	14	41.2	54	54.5
Moderate	11	32.4	27	27.2
Moderate-poor	2	5.9	4	4.0
Poor	7	20.6	10	10.1
Missing	0	0	1	1
**Stage**				
Stage 1	0	0	0	0
Stage 2	11	32.4	44	44.2
Stage 3	23	67.6	54	54.5
Stage 4	0	0	0	0
Missing	0	0	1	1
**T-stage**				
T1	0	0	0	0
T2	0	0	10	10.1
T3	14	41.2	50	50.5
T4	20	58.8	38	38.3
Missing	0	0	1	0
**N-Positivity**				
N0	11	32.4	44	44.5
N1	23	67.6	55	55.5
Missing	0	0	0	0
**ZEB2 Positive**				
Yes	24	70.5	49	49.5
No	10	29.5	50	50.5

Table 2: Clinical and pathological parameters of patients in the pilot and validation study and their association with nuclear ZEB2 expression. *p*-values were derived using Chi squared or Fisher's exact tests.

	**Pilot study**			**Validation cohort**		
**Characteristics**	**ZEB2+VE**	**ZEB2-VE**	***p-value***	**ZEB2+VE**	**ZEB2-VE**	***p-value***
**Age**						
**<60**	1	1	***p=0.51***	14	10	***p=0.60***
**>60**	23	9		36	39	
**Sex**						
**M**	10	6	***p=0.46***	24	29	***p=0.40***
**F**	14	4		25	21	
**pT-stage**						
**T1**	0	0	***p=0.15***	0	0	***p=0.30***
**T2**	0	0		5	5	
**T3**	8	6		27	23	
**T4**	16	4		18	21	
**pN status**						
**N0**	8	3	***p=0.85***	19	25	***p=0.30***
**N1/N2**	16	7		30	25	
**AJCC Stage**						
**1**	0	0	***p=0.17***	0	0	***p=0.25***
**2**	8	3		18	26	
**3**	16	7		31	23	
**4**	0	0		0	0	
**Differentiation**						
**Well**	0	0	***p=0.64***	2	1	***p=0.84***
**Mod-well**	10	4		26	28	
**Moderate**	9	2		13	14	
**Mod-poor**	1	1		3	1	
**Poor**	4	3		5	5	

Table 3: Methodological detail of the biomarker study reported in accordance with REMARK guidelines.

**REMARK guidelines for biomarker reporting**	
Biomarker examined	ZEB2
Disease studied	Colorectal adenocarcinoma
Database	Prospective
Time period	2005-2013
Hypothesis	ZEB2 expression prognosticates poor patients' survival after adjuvant FOLFOX chemotherapy
Inclusion criteria	Primary colorectal adenocarcinoma + Surgical resection
Exclusion criteria	Synchronous metastasis at presentation
Treatment	Surgical resection + FOLFOX chemotherapy
Biological material	Paraffin embedded human tissue
Biomarker detection	Automated Immunohistochemistry / Leica XL Autostainer
Antibody	In-house/Rabbit/Polyclonal/1:750 dilution
Quality control	Antibody optimisation on uterine myometrium<br>Positive Control – Fibroblasts / Uterine tissue / Tonsil<br>Negative control – Normal colon
Scoring	2 independent pathologists blinded to the clinical details/Nuclear ZEB2 staining/>10% of cancer cells /Positive or Negative
Median follow up:	
Pilot study	
Validation study	36 months<br>42 months
Clinical end points	Overall survival – time to death (Clinical records) from date of surgery<br>Disease free survival – Radiological detection of recurrence from date of surgery.<br>Distant recurrence – Radiological detection of metastasis outside the colon/rectum after surgery<br>Local recurrence – Radiological evidence of recurrence in the colon or rectum after surgery
Cox regression model	Age, T stage, Node positivity, Differentiation, ZEB2,

Table 4: Multivariate analysis (Cox proportional hazard regression model) of prognostic parameters for overall survival in colorectal cancer patients who received adjuvant FOLFOX therapy.

Characteristic	HR	95% CI	*p* - value
**Age (<60 vs. >60)**	1.2	0.48 – 3.24	0.66
**T-stage (Overall)**			0.01
**T stage (T1/2 vs. T4)**	7.01	1.60 – 30.89	0.01
**T stage (T3 vs. T4)**	3.10	0.69 – 13.67	0.15
**N-stage (N0 vs. N1/2)**	2.10	1.60 – 6.17	0.03
**Differentiation**			0.18
**Differentiation (Well vs. Poor)**	1.29	0.58 – 2.85	0.537
**Differentiation (Mod vs. Poor)**	1.04	0.23 – 1.63	0.33
**ZEB2 Status (pos vs. neg)**	3.13	1.59 - 6.16	0.001

Table 5: Multivariate analysis (Cox proportional hazard regression model) of prognostic parameters for disease free survival in colorectal cancer patients who received adjuvant FOLFOX therapy.

Characteristic	HR	95% CI	*p* - value
**Age (<60 vs. >60)**	1.91	0.738 – 4.98	0.19
**T-stage (Overall)**			0.006
**T stage (T1/2 vs. T4)**	7.03	1.53 – 32.25	0.01
**T stage (T3 vs. T4)**	2.29	0.50- 10.50	0.29
**N-stage (N0 vs. N1/2)**	2.04	1.02 – 4.12	0.05
**Differentiation**			0.19
**Differentiation (Well vs. Poor)**	0.97	0.430 -2.16	0.92
**Differentiation (Mod vs. Poor)**	0.46	0.17 – 1.23	0.12
**ZEB2 Status (pos vs. neg)**	3.12	1.53 – 6.65	0.002

Table 6: Clinical and pathological parameters of patients with primary colorectal cancer and matched colorectal liver metastases.

	n	%
**Age (Yrs)**		
<60	8	26.7
>60	22	73.3
**Site of tumour**		
Colon	14	46.7
Rectum	16	53.3
**pT stage**		
1	0	0
2	3	10
3	21	70
4	6	20
**pN-Positivity**		
N0	12	40
N1/2	18	60
**Metastasis at presentation**		
MO	12	40
M1	18	60
**Stage**		
Stage 1	0	0
Stage 2	4	13.3
Stage 3	8	26.7
Stage 4	18	60
**Synchronous vs. metachronous**		
Synchronous	12	40
Metachronous	18	60
**Differentiation**		
Well	0	0
Moderate-well	9	30
Moderate	18	60
Moderate-poor	0	0
Poor	1	3.3
Missing	2	6.7
**Neo Adjuvant Chemotherapy**		
Yes	11	63.3
No	19	36.6
**ZEB2 Positive primary**		
Yes	26	86.6
No	4	13.4
**ZEB2 positive Liver metastasis**		
Yes	25	83.3
No	5	16.7

Table 7: Primers used in the study

Name	Sequence (5’-3’)	Product Size	Purpose	Accession #
**GAPDH-F**	GGCTGAGAACGGGAAGCTTGTCAT	143 bp	qPCR, PCR	NM_002046
**GAPDH-R**	CAGCCTTCTCCATGGTGGTGAAGA			
**CDH1-F**	GCTGGACCGAGAGAGTTTCC	155 bp	qPCR	NM_001317 184
**CDH1-R**	CAAAATCCAAGCCCGTGGTG			
**Vim-F**	CTCTGGCACGTCTTGACCTT	231 bp	qPCR	NM_003380
**Vim-R**	ACCATTCTTCTGCCTCCTGC			
**ZEB2-F**	AAGATAGGTGGCGCGTGTTT	752 bp	qPCR	NM_014795
**ZEB2-R**	CTGGCCCCATAGTGTCATAGTC			
**Pol II-F**	Not disclosed by supplier (Active Motif)	180 bp	ChIP	N/A
**Pol II-R**	Not disclosed by supplier (Active Motif)			
**ERCC1-qF**	CCTTGAGGCTCCAAGACCAG	193 bp	qPCR	NM_001983
**ERCC1-qR**	GGAATAAGGGCTTGGCCACT			
**ERCC1-F**	ATGGACCCTGGGAAGGAC	822 bp	PCR	NM_001983
**ERCC1-R**	TCAGGGTACTTTCAAGAAGGG			
**ERCC1-P-Forward 1**	TAGGAGCTCTTGGTCAACTTGAGACAATTGG	1554 bp	Promoter/ Cloning	ERCC1-202 ENST00000300853.7
**ERCC1 Reverse**	TGTAAGCTTACATTGACTTGGCTTCAGTTTCCTC			
**ERCC1-P-Forward 2**	TAGGAGCTCTCAGAACGGAACGGGATTGATAAATAG	667 bp	Promoter / Cloning	
**ERCC1-F1 EBOX 1-4**	ACCAAGTTGGATCTCCTGCG	435 bp	PCR/ ChIP	ERCC1-202 ENST00000300853.7
**ERCC1-R1-EBOX 1-4**	TCCATCTCTCAGACTCGGCA		PCR/ChIP	

**CDH1-F**<br>**E-box 4-5**	ACCCTAGCAACTCCAGGCTA	224 bp	PCR/ChIP	CDH1-201<br>ENST000002<br>61769.9
**CDH1-R E-**<br>**E-box 4-5**	CAAGCTCACAGGTGCTTTGC		PCR/ChIP	
**CDH1-F**<br>**E-box 1-3**	GTAATCCAACACTTCAGGAG	524	PCR/ChIP	CDH1-201<br>ENST000002<br>61769.9
**CDH1-R**<br>**E-box 1-3**	GCCTCTCTAGTAGCTGGGAG		PCR/ChIP	
**ERCC1-F1**<br>**EBOX 5-7**	CTTACATAGATTAAAGCCAG	356	PCR/ ChIP	ERCC1-202<br>ENST000003<br>00853.7
**ERCC1-R1-**<br>**EBOX 5-7**	CGATTCTCCTGCCTCCTTAG		PCR/ChIP	

3. Supplementary Figures

Supp. Fig. 1

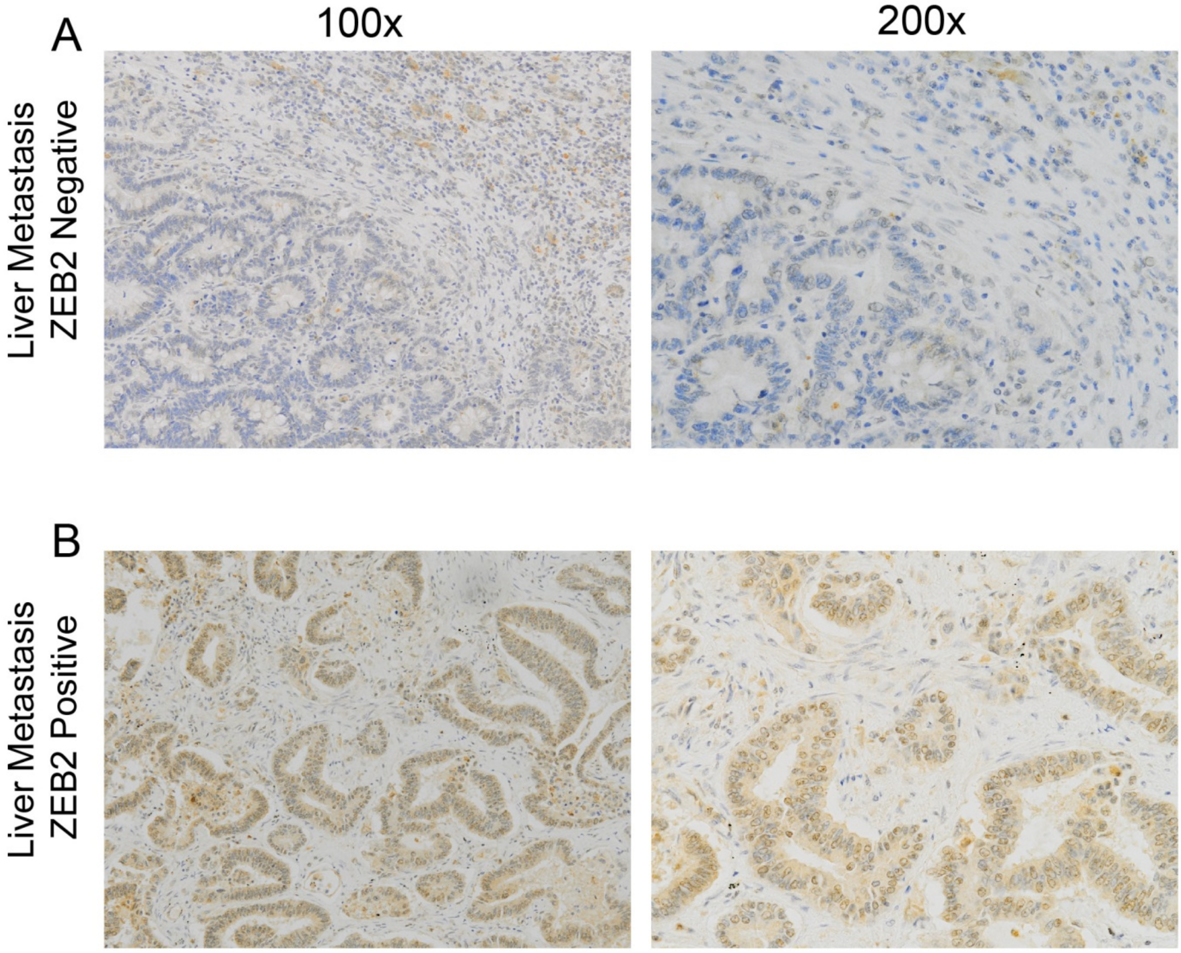

Supplementary Figure 1: ZEB2 expression in CRC-liver metastasis. ZEB2 expression was analysed in a cohort of 30 CRC patients with synchronous/metachronous metastases paired with primary tumours. Images illustrate ZEB2-negative **(A)** and -positive **(B)** cases.

Supp. Fig. 2

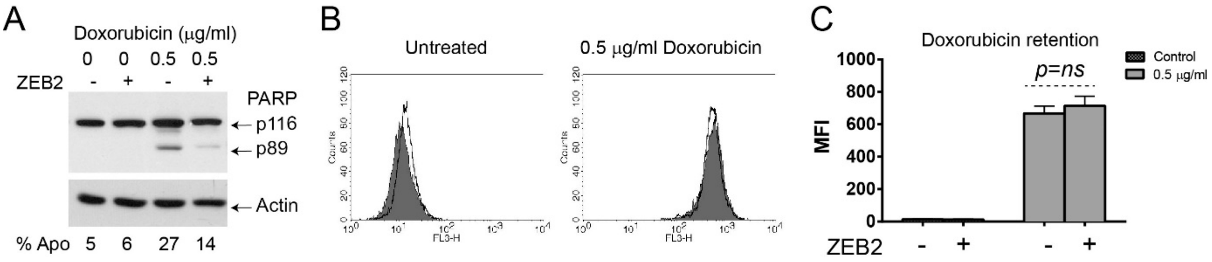

Supplementary Figure 2: ZEB2 has no effect on drug efflux in DLD1 cells. (A) ZEB2 reduces doxorubicin-induced apoptosis in DLD1cells. Cells were cultured with or without DOX for 3 days and treated with 0.5μg/ml doxorubicin for 24 hours. Apoptosis was assessed using Annexin V/PI staining (presented as % apo) and PARP cleavage. ZEB2-expressing cells were less sensitive to doxorubicin than ZEB2-negative counterparts. **(B)** To investigate drug efflux, un-induced and induced DLD1-ZEB2 cells were incubated with doxorubicin for 1 hour, washed and analysed 4 hours later using flow cytometry to detect doxorubicin retained in the cells. Both un-induced (ZEB2-) and induced (ZEB2+) cells exhibited similar fluorescence emission **(B)** and median fluorescence intensity **(C)** upon doxorubicin treatment suggesting that ZEB2 does not affect the quantity of intracellular doxorubicin.

Supp. Fig. 3

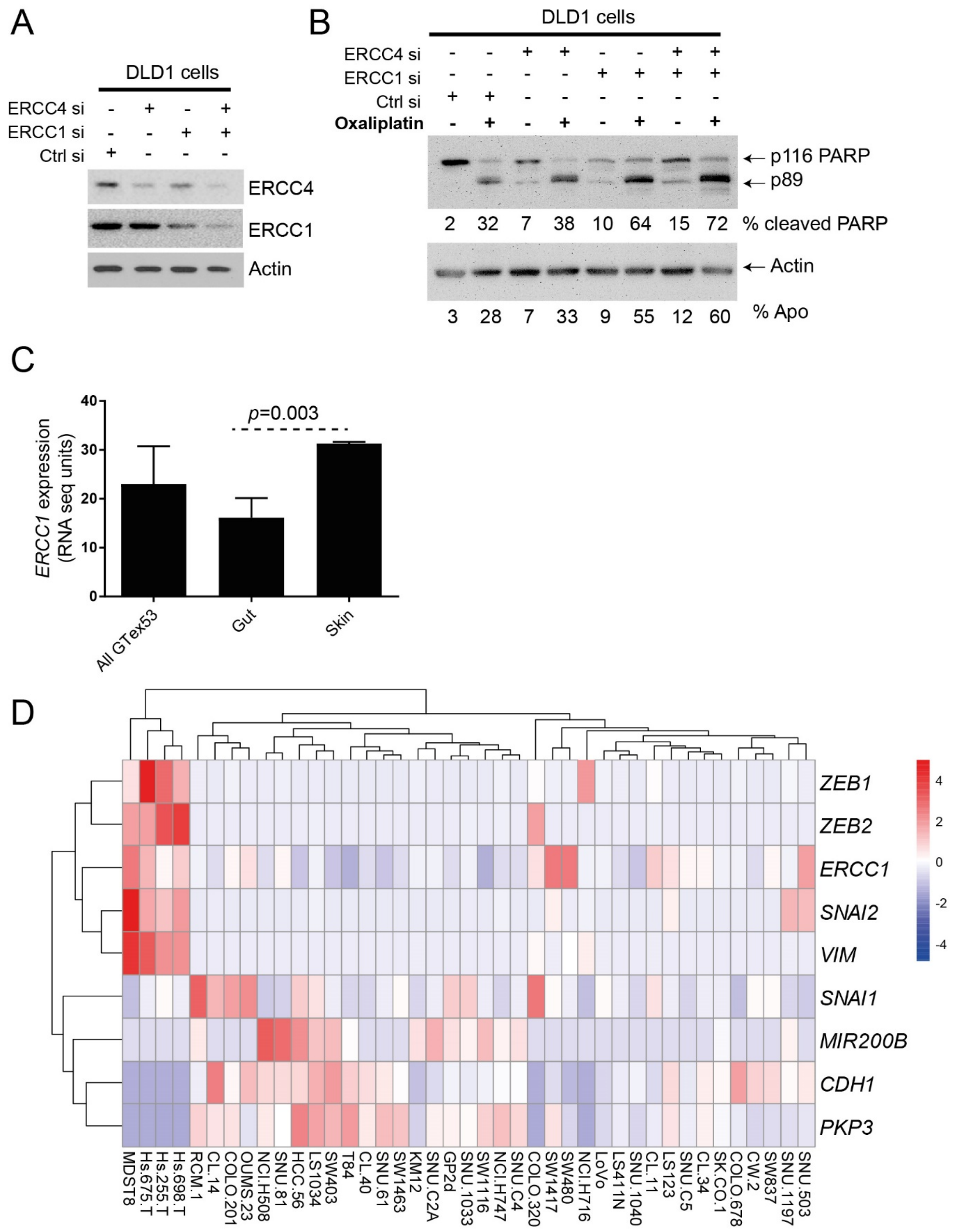

Supplementary Figure 3: Contribution of ERCC1 and ERCC4 to oxaliplatin response. (A) Small interfering RNA for *ERCC1*, *ERCC4* or both were transfected to DLD cells to understand their relative contribution to oxaliplatin resistance. All siRNAs worked to substantially reduce their target gene expression as assessed by western blotting. **(B)** DLD cells with reduced ERCC1 and/or ERCC4 expression were subjected to 100mM oxaliplatin for 16

hours and apoptosis was assessed using PARP cleavage and Annexin V externalisation (% Apo). *ERCC1* alone and *ERCC1+ERCC4* knock down caused a substantial sensitisation to oxaliplatin induced apoptosis. **(C)** *ERCC1* expression was analysed in normal tissues represented in the GTEx53 cohort of Gene Atlas database (1). *ERCC1* is ubiquitously expressed in all tissues, but expression in the skin is 2-fold higher than that in colon and other internal organs of the digestive system, namely oesophagus, stomach, duodenum, small intestine, large intestine, liver and pancreas. **(B)** The CRC cell lines in the CCLE cohort of Gene Atlas database (1) were probed for the expression of *ZEB1*, *ZEB2*, *SNAI1*, *SNAI2*, *Vimentin* (mesenchymal markers), *mir200B*, *PKP3* and *CDH1* (epithelial markers) and *ERCC1*. Note that *ERCC1* expression tends to correlate with the EMT status and ZEB2^{high}/*CDH1*^{low} pattern, therefore can be considered as a part of mesenchymal gene expression signature.

Supp. Fig. 4

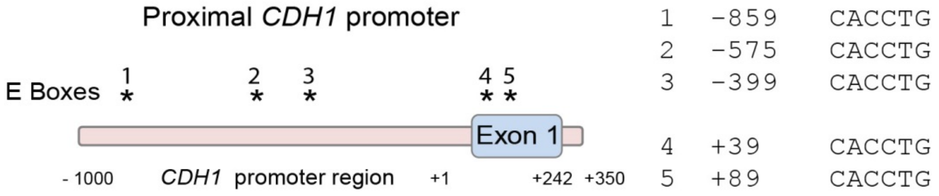

Supplementary Figure 4: The promoter and regulatory region of *CDH1* gene and E-box elements listed (on the right) and presented graphically (on the left) with respect to transcription start site.

Supp. Fig. 5

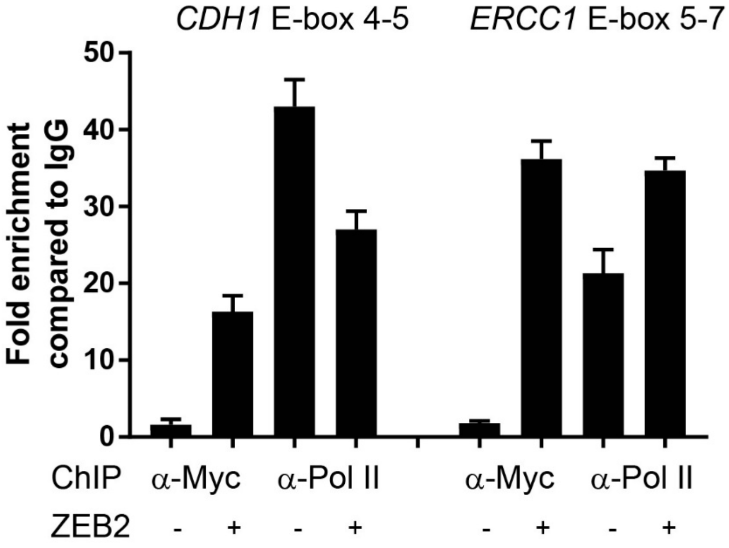

Supplementary Figure 5: The enrichment of E-boxes in the promoter of *CDH1* (E-box 4-5) and the E-box cluster (E-box 5-7) of *ERCC1* genes were analysed using qPCR upon ChIP performed using c-MYC-tag (ZEB2) or RNA pol-II (Pol II) antibodies in DLD1-ZEB2 cells. As the binding of ZEB2 increased, Pol II binding to *CDH1* promoter decreased indicating repression. However, when ZEB2 is expressed (ZEB2+), *ERCC1* E-box 5-7-containing chromatin was enriched for both c-MYC-ZEB2 and Pol II suggesting that ZEB2 binds to this locus and activates *ERCC1* gene expression.

Supp. Fig. 6

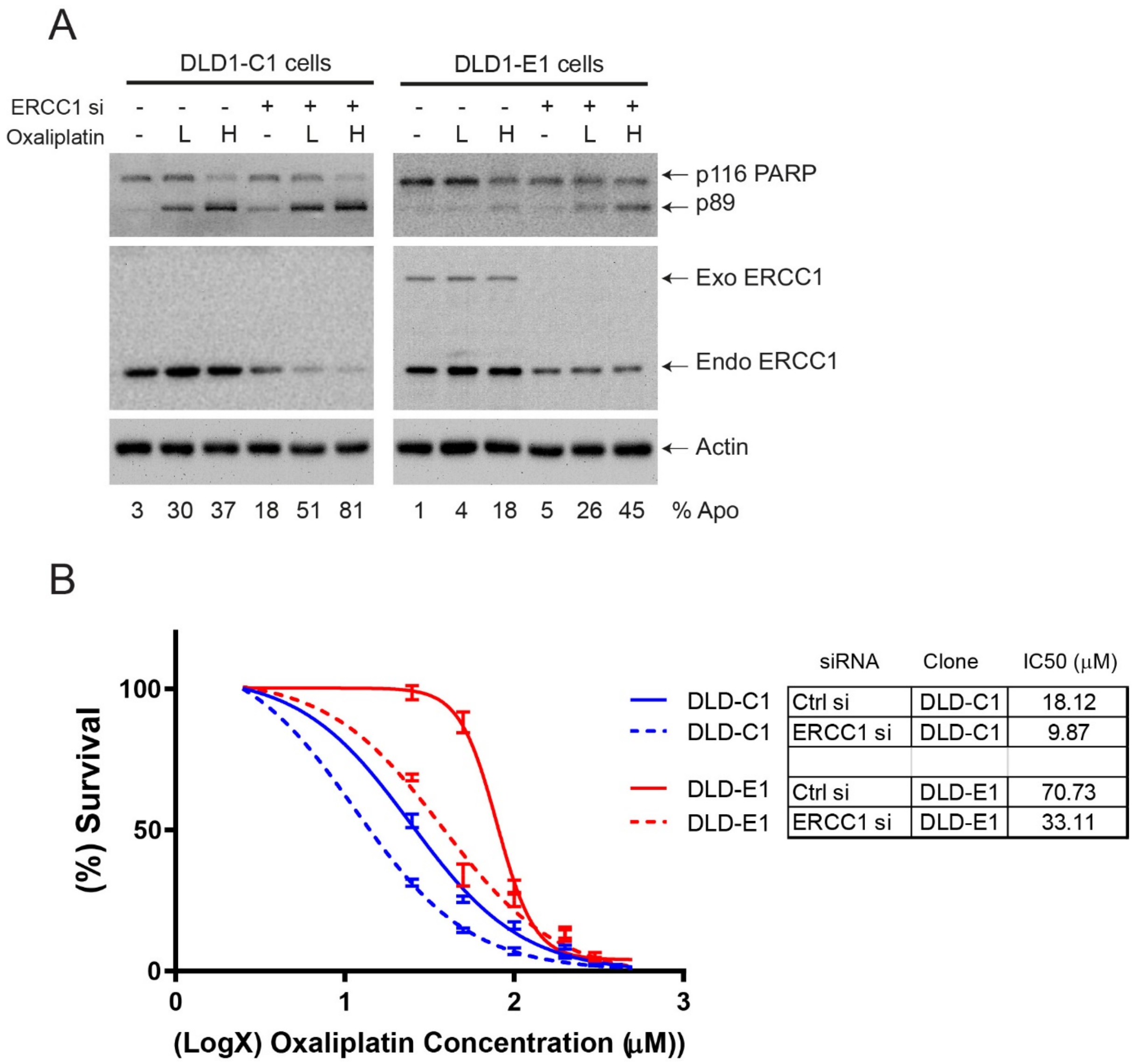

Supplementary Figure 6: ERCC1 overexpression but not clonal selection is responsible for oxaliplatin resistance of DLD clones. (A) Control siRNA (ERCC1 si -) or *ERCC1* siRNA (ERCC1 si +) was transfected to DLD-C1 or DLD-E1 cells. ERCC1 blot shows the downregulation of both exogenous and endogenous proteins. These cells were incubated with low (100mM, L) or high (200mM, H) oxaliplatin for 16 hours. Apoptosis was assessed using PARP cleavage and Annexin V externalisation (% Apo). **(B)** Viability assay assessing oxaliplatin response in *ERCC1* depleted DLD-C1 and DLD-E1 cells show a decreased *IC50* value cells as presented with graph (left) and table (right).

Supp. Fig. 7

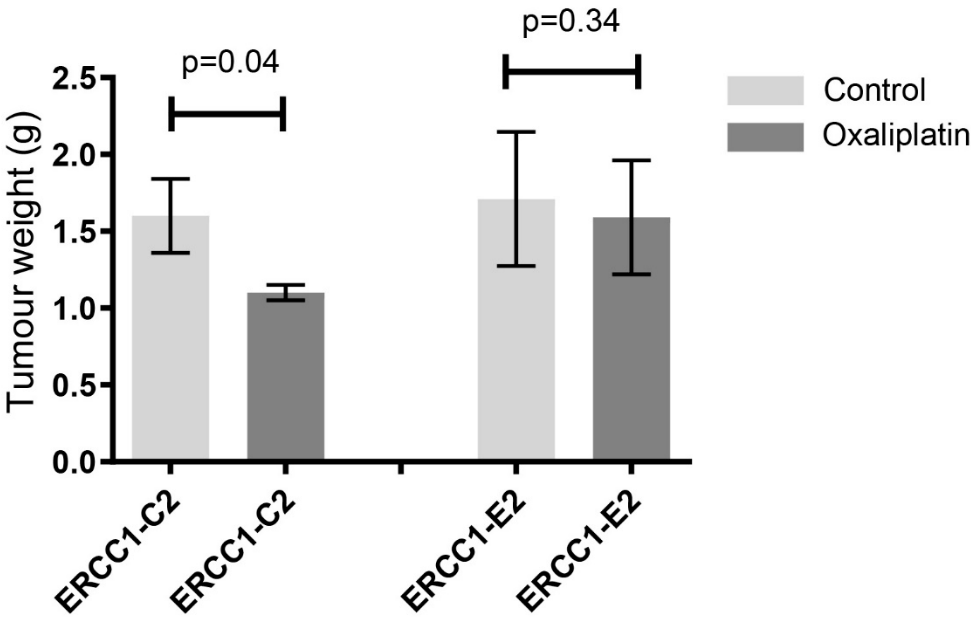

Supplementary Figure 7: Weight of tumours obtained from DLD-C2 or DLD-E2 cells, with or without oxaliplatin treatment, were assessed. ERCC1 overexpressing cancers did not show a significant reduction of tumour weight upon chemotherapy.

4. References

1. Petryszak R, Keays M, Tang YA, Fonseca NA, Barrera E, Burdett T, et al. Expression Atlas update--an integrated database of gene and protein expression in humans, animals and plants. Nucleic Acids Res. 2016;44(D1):D746-52.
